# Identification and verification of genes associated with hypoxia microenvironment in Alzheimer’s disease

**DOI:** 10.1038/s41598-023-43595-9

**Published:** 2023-09-27

**Authors:** Mingyang Yuan, Yanjin Feng, Mingri Zhao, Ting Xu, Liuhong Li, Ke Guo, Deren Hou

**Affiliations:** 1 The Third Xiangya Hospital, Department of Neurology, Central South University, Changsha, 410000 China; 2 School of Life Sciences, Center for Medical Genetics and Hunan Key Laboratory of Medical Genetics, Central South University, Changsha, 410000 China

**Keywords:** Computational biology and bioinformatics, Diseases, Neurology, Risk factors

## Abstract

As the incidence of Alzheimer's disease (AD) increases year by year, more people begin to study this disease. In recent years, many studies on reactive oxygen species (ROS), neuroinflammation, autophagy, and other fields have confirmed that hypoxia is closely related to AD. However, no researchers have used bioinformatics methods to study the relationship between AD and hypoxia. Therefore, our study aimed to screen the role of hypoxia-related genes in AD and clarify their diagnostic significance. A total of 7681 differentially expressed genes (DEGs) were identified in GSE33000 by differential expression analysis and cluster analysis. Weighted gene co-expression network analysis (WGCNA) was used to detect 9 modules and 205 hub genes with high correlation coefficients. Next, machine learning algorithms were applied to 205 hub genes and four key genes were selected. Through the verification of external dataset and quantitative real-time PCR (qRT-PCR), the AD diagnostic model was established by ANTXR2, BDNF and NFKBIA. The bioinformatics analysis results suggest that hypoxia-related genes may increase the risk of AD. However, more in-depth studies are still needed to investigate their association, this article would guide the insights and directions for further research.

## Introduction

Alzheimer's disease (AD), the most common form of progressive neurodegenerative disorder, is characterized by memory loss and cognitive deterioration. Epidemiological studies have confirmed that there were 57.4 million AD patients worldwide in 2019^1^. The number is expected to reach 152 million by 2050. Effective treatment options for AD are limited, including cholinesterase inhibitors, N-methyl-D aspartic acid receptor antagonists, and drugs that target beta-amyloid (Aβ). However, no utterly effective cure can stop or reverse the progression of the disease^2^. Due to the heavy economic and mental burden on society and family, AD is emerging as a significant health challenge. Therefore, it is urgent to strengthen the research on the etiology and pathogenesis of AD. It is generally considered that the occurrence of AD is related to the combination of genetic and environmental factors^3^. However, the detailed molecular mechanisms underlying these factors remain unclear^4^. Recently, scientists have identified several significant factors, such as cerebral ischemia^5^, oxidative stress, neuroinflammation^6^, and others, which may contribute to AD onset and progression.

Hypoxia is a pathological process caused by insufficient oxygen supply or lack of oxygen use. It may lead to abnormal changes in tissue metabolism, function, and morphological structure^7^. Although the brain's weight only accounts for 2%-3% of the total body weight, the oxygen consumption of the brain tissue accounts for more than 20%-30% of the total oxygen consumption. Without the ability to store oxygen, the brain relies entirely on the oxygen the blood carries to maintain normal physiological functions. Thus, it becomes the most sensitive one to hypoxia among all the organs^8^. Lack of sufficient oxygen can influence brain cell functions and have long-term effects on neurological function^9,10^.

Various studies have indicated that the pathogenesis of AD is closely related to hypoxia. Hypoxia can up-regulated BACE1 gene expression and Aβ production^11^. As for Aβ clearance, further research has suggested that hypoxia can reduce the expression of enkephalin (NEP)^12,13^, which was the major degrading enzyme of Aβ to decrease the Aβ deposition^14^. Hypoxia may have numerous pathological consequences, such as the production of reactive oxygen species (ROS), dysregulation of calcium homeostasis, activation of microglia, and other neuroinflammatory responses. These consequences indirectly promote Aβ toxicity and further aggravating neuronal death^15,16^. The brain would induct the expression of hypoxia-inducing factor 1 (HIF-1) to adapt to a hypoxia situation through the conventional adaptive mechanisms. HIF-1 is the principal molecule regulating expressions of hypoxia-responsive genes, which would play a crucial role in cellular adaptation to low oxygen levels^17^. When cells experience hypoxia, HIF-1 is stabilized and activated through the inhibition of an enzyme called prolyl hydroxylase 2 (PHD2), leading to increased HIF-1 gene expression^18^. HIF-1 can regulate glucose metabolism, cerebral blood flow and erythropoiesis to reduce hypoxic brain injury. Currently, there is a growing body of evidence suggesting the involvement of HIF-1 in various diseases related to some central nervous system (CNS), including neurodegenerative disorders, stroke and others^19^. However, severe or prolonged hypoxia has the ability to convert HIF-1 into a stimulator of cellular processes that have negative effects, resulting in the production of Aβ and cell death^20^. Scientists have demonstrated that the downregulation of HIF-1 may alleviate tau pathology and cognitive impairment^21^. Previous studies have shown that oxygen therapy can improve AD's pathological aspects and clinical symptoms^22^. Therefore, it is reasonable to believe that hypoxia plays an essential role in the occurrence and development of AD. There have been no reports on gene databases to introduce the relationship between AD and hypoxia. Identifying and verifying the genes related to hypoxia in AD patients using bioinformatic methods may be significant to reveal the pathogenesis of AD.

In our study, data acquired from Gene Expression Omnibus (GEO) and Molecular Signatures Database (MsigDB) were analyzed to filter the hypoxia genes related with A.D. The genes obtained from the difference analysis were analyzed by co-expression network analysis (WGCNA). After screening related module genes, we used Gene Ontology (GO) and Kyoto Encyclopedia of Genes and Genomes (KEGG) to evaluate the possible biological functions of Hub genes. To further improve the ability of hypoxia-related genes to predict AD, we adopted multiple machine learning methods for feature screening. We identified four genes that were related to hypoxia and AD, and constructed ROC curves and NOR from internal and external data sets to evaluate their predictive efficacy. qRT-PCR was performed on the hippocampus of AD mice and the same brain parts of wild-type mice. Except that the expression level of MOV10L1 gene was too low to be detected, the expression trend of the other three genes (NFKBIA, ANTXR2, BDNF) was consistent with the verification results of internal and external datasets. Finally, the hippocampus of APP/PS1 and wild-type mice at six months of age were tested to confirm the predictive efficacy of these four genes.

## Results

### Identification of differentially expressed genes

**Figure 1:**
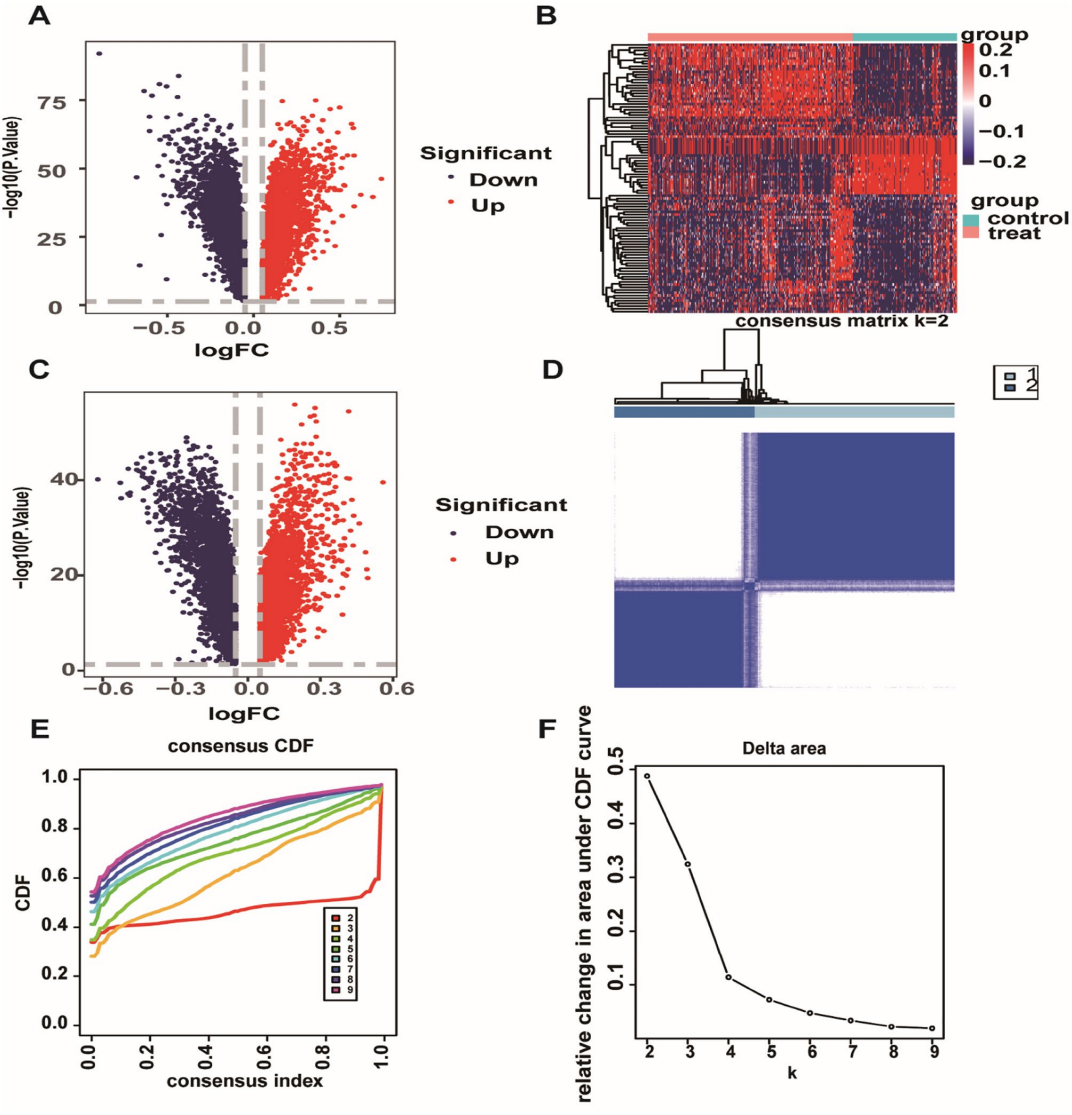
Exploration of hypoxia-associated genes. (**A**,**B**) Volcano maps and heatmap to display Differential Genes. (P-value < 0.05, | (log2FC) |> 0.05). Using this screening criterion, 3074 genes were identified as up-regulated and 3,198 genes as down-regulated. At the same time, the differential genes of the results GSE33000 data can be seen from figure B, and the difference is very obvious. (**C**) Results of the difference analysis between "hypoxia-low" and "hypoxia-high". (**D**–**F**) CDF curve and the Delta area curve of consensus clustering. Using R to cluster between AD patients, when the number of clusters is 2, the discrimination is the highest and the clustering effect is the best.

In R software, 3,074 genes were identified as up-regulated and 3,198 genes down-regulated through difference analysis of GSE33000 dataset. The results are presented in volcano (Fig. 1A) and heatmaps (Fig. 1B). Shown is the difference between the top 100 genes with the most significant difference between AD and normal groups according to GSE33000. Using unsupervised clustering based on the dataset of hypoxia hallmark genes, we found that when k = 2, the classification was highly reliable and sturdy (Fig. 1C–F). The 310 AD samples in the GSE33000 dataset were divided into “hypoxia-low" and "hypoxia-high" groups with 182 samples for subtype 1 and 128 for subtype 2. In the two subtypes, the DEGs are presented in the volcano plot. The results of difference analysis in "hypoxia-low" and "hypoxia-high" suggested that 3011 genes were up-regulated and 3,046 genes down-regulated. Merge 6272 DEGs which gain from the AD group and the normal group with 6057 DEGs in "hypoxia-low" and "hypoxia-high", 7681 DEGs were finally obtained.

### Identification hub genes by WGCNA

**Figure 2:**
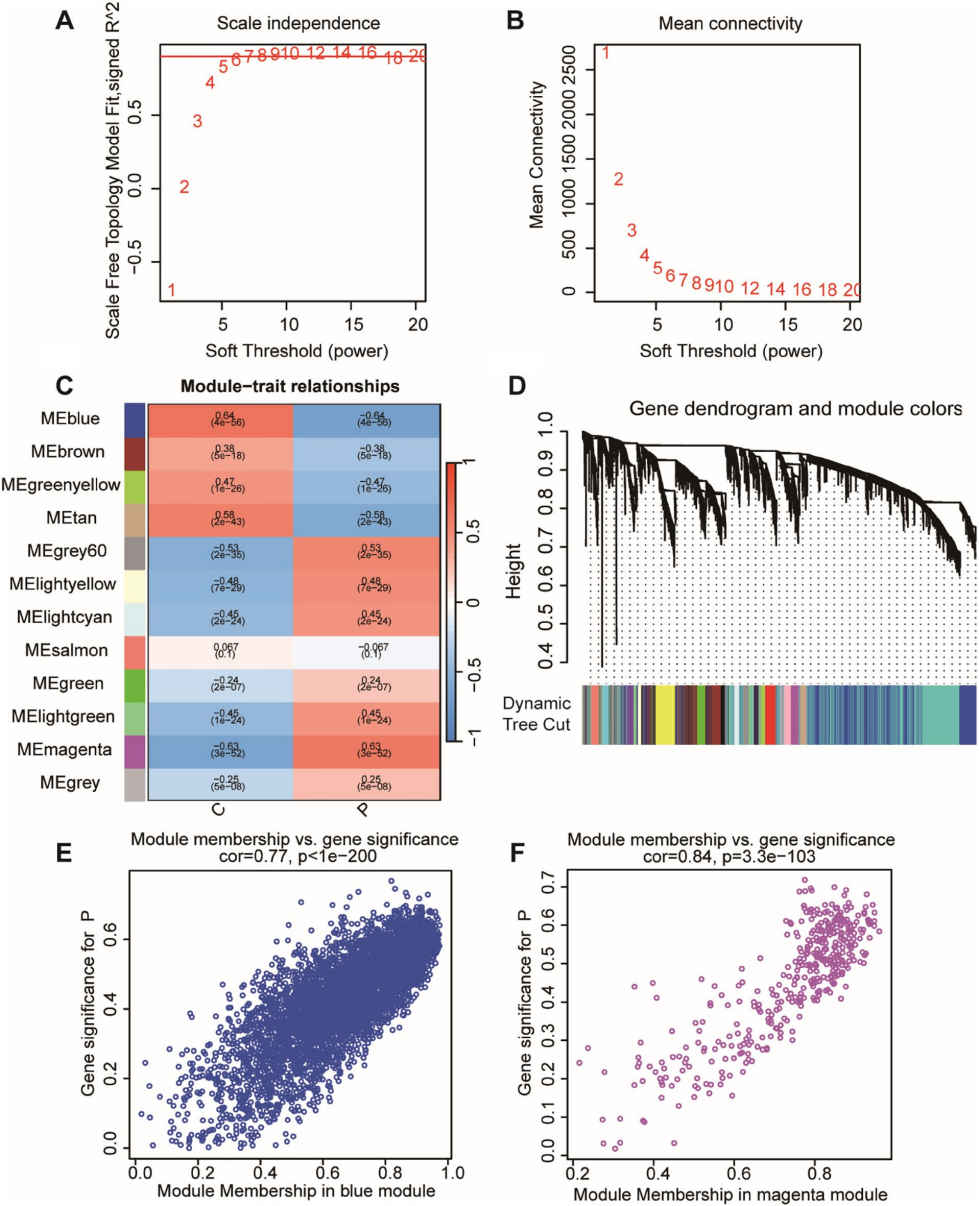
Construction of WGCNA co–expression network. (**A**,**B**) The scale-free fit index and mean connectivity of WGCNA. It exhibits the soft thresholding power β in the WGCNA. The x-axis represents the soft-threshold power. Based on proportional independence and average connectivity analysis, β = 7 was selected as the soft threshold to construct the network. (**C**,**D**) The DEGs genes of hypoxia divided into nine modules. Panel C shows the gene tree showing the different gene co-expression modules (each module is indicated by a different unique color). (**E**,**F**) Scatterplots of GS and MM in the MEblue and MEmagenta modules. Scatter plots of correlation analysis between gene membership status and gene importance within modules are shown.

We used 7681 DEGs genes via WGCNA to further identify hypoxia-related genes. Based on the results of R, when the co-expression network was built, we observed that the soft thresholding power β was 7 (scale-free *R*^2^ = 0.8) (Fig. 2A,B). It was found that nine modules were certified by the average linkage hierarchical clustering and the soft thresholding power (Fig. 2C,D). C represents the control group, and P represents the AD patients. We find that the MEblue module (cor = 0.77, p < 1e − 200) and the MEmagenta module (cor = 0.84, p < 3.3e − 103) were most relevant with hypoxia genes (Fig. 2E,F). In these two modules, a total of 205 genes were selected using the screening criteria of set | GS |> 0.62 and| MM |> 0.82.

### GO and KEGG enrichment analysis of hub genes

**Figure 3:**
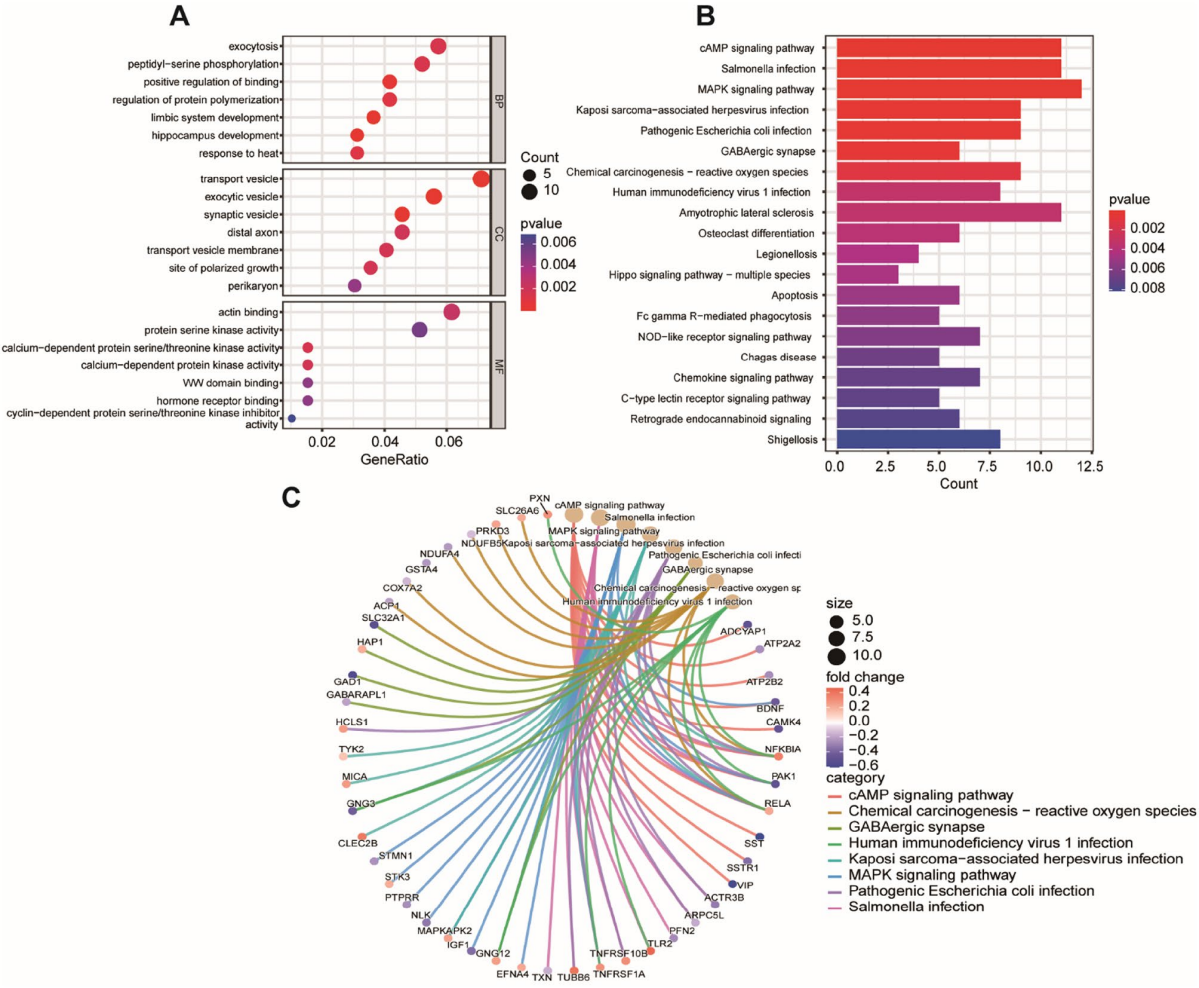
The result of the GO and KEGG. (**A**) Scatter plot of top 20 KEGG pathways (**B**) Scatter plot of top 7 enriched GO terms of molecular function (MF), biological process (BP), and cellular component (CC). (**C**) The genes most relevant to the first six pathways.

We selected the most significantly (p < 0.05) enriched pathways through GO and KEGG. The hub genes were enriched in biological processes, including exocytosis, peptidyl − serine phosphorylation, and positive binding regulation (Fig. 3A). The cell components of hub genes were gathered with transport vesicle, exocytic vesicle, and synaptic vesicle. The analysis of molecular function was significant (p < 0.05) in the actin binding, protein serine kinase activity, and calcium − dependent protein serine/threonine kinase activity (Fig. 3B,C). The bar chart presents the TOP 20 KEGG pathways and the TOP 6 critical KEGG pathways of hub genes enriched in MAPK signaling pathway, pathways of neurodegeneration-multiple diseases, cAMP signaling pathway, Salmonella infection, Amyotrophic lateral sclerosis, and Alzheimer's disease.

### AD-related genes classifier screened by machine learning

**Figure 4:**
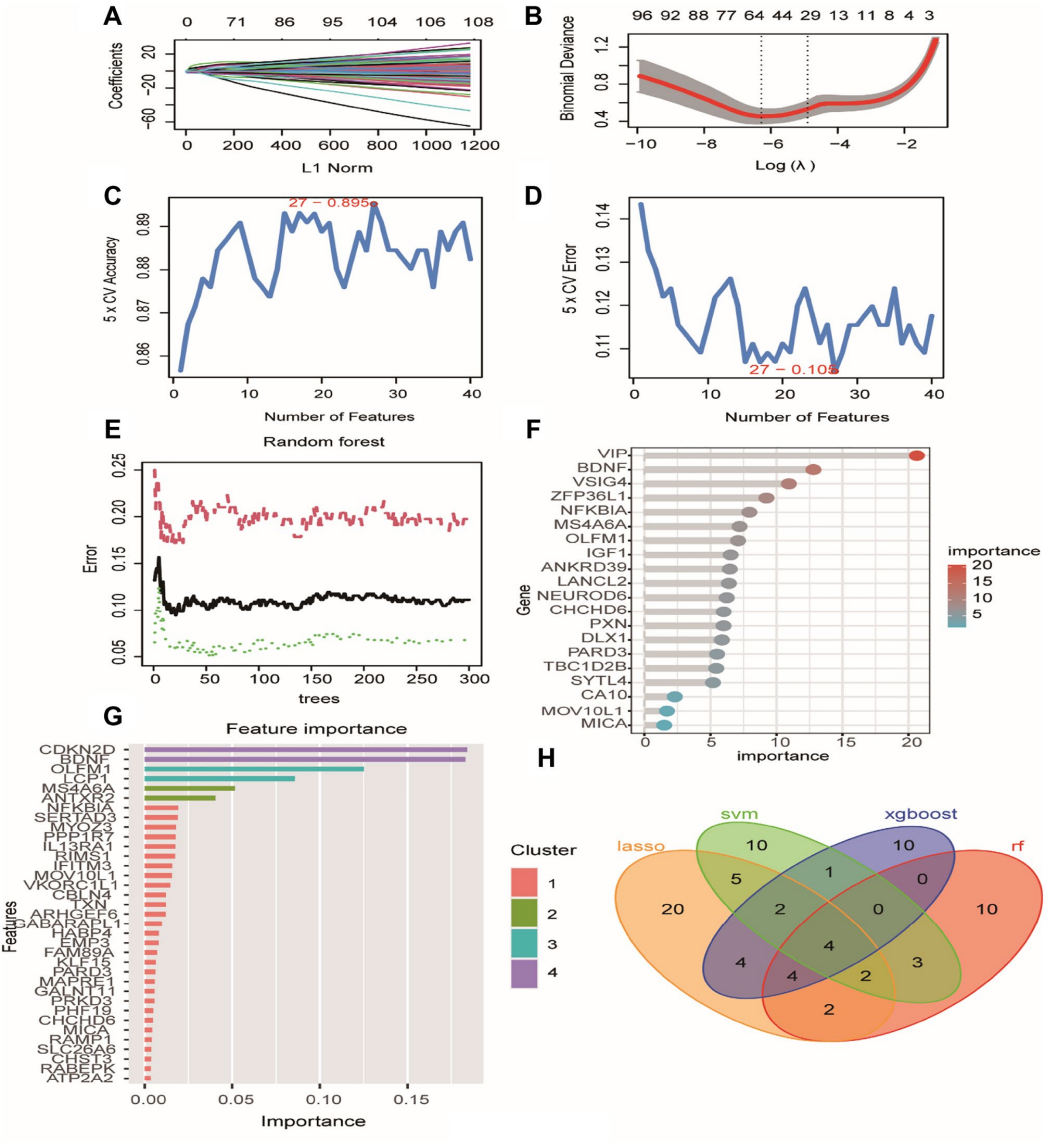
Screening of signature genes. (**A**) Distribution of key gene LASSO regression coefficients. (**B**) The suitable Log (Lambda) value in the penalty item parameter diagram of the LASSO model has been selected and get further analysis. Screening of AD diagnostic biomarkers based on LASSO model (the optimal number of genes was the lowest point of the curve) (**C**,**D**) Choose the Maximum accuracy and minimum error to construct the module in SVM-RFE. (**E**,**F**) The most important 27 hub genes identified by RF. (**G**) XGBoost algorithm verified the materiality of the features and extracted the top 25 parameters. (**H**) Overlapping genes in LASSO, SVM, RF, and XGBoost. Through Venn diagram, the intersection of the four machine learning results was taken, and finally four genes were identified as the marker genes between AD and hypoxia.

In LASSO algorithm, 43 genes were screened for the next operation through characteristic variables (Fig. 4A,B). The number of features of the 205 genes previously identified by WGCNA were identified by SVM-RFE. When the number of genes dropped to 27, the accuracy was the highest and the error rate was the lowest (Fig. 4C,D). Random forest algorithm and XGboost algorithm screened the top 25 genes with the highest weight as marker genes (Fig. 4E–G). Venn algorithm was used to select four cluster features (Fig. 4H).

### Single gene set enrichment analysis of marker gene

**Figure 5:**
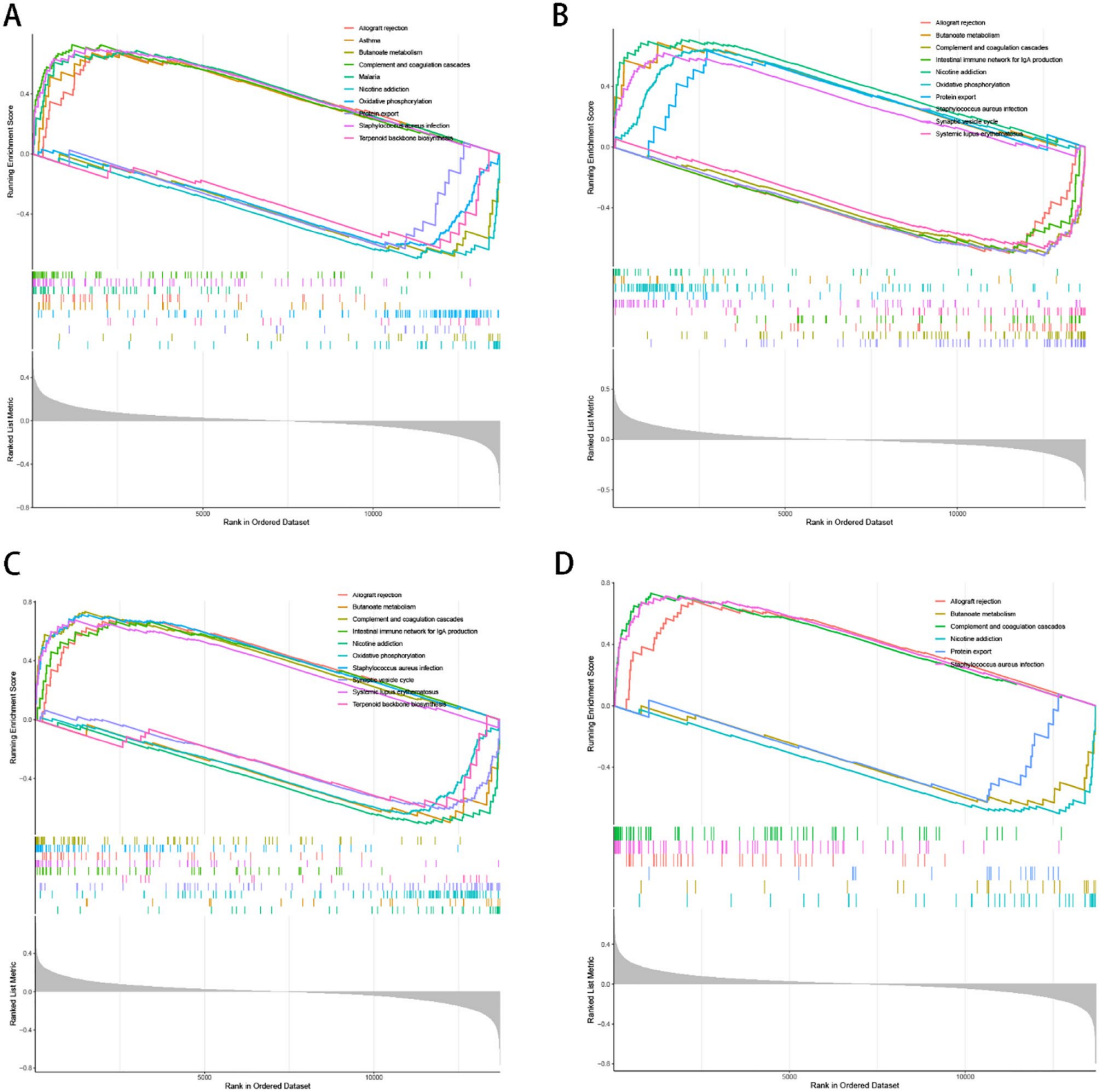
Outcomes of the Single gene set enrichment analysis. (**A**–**D**) Single gene set enrichment analysis (GSEA) of four marker genes. The enrichment results of the four genes indicated the significance of hypoxia in AD.

The GSEA results of the four genes ("ANTXR2", "BDNF," "NFKBIA", "MOV10L1") enriched in Oxidative phosphorylation, Allograft rejection, complement and coagulation cascade pathways and so on. At the same time, these genes were enriched through precisely the pathways associated with AD formation. It also suggests that the screened genes may play an important role in the occurrence and development of AD. (Fig. 5A–D).

### Evaluation of the Alzheimer's disease diagnosis model

**Figure 6:**
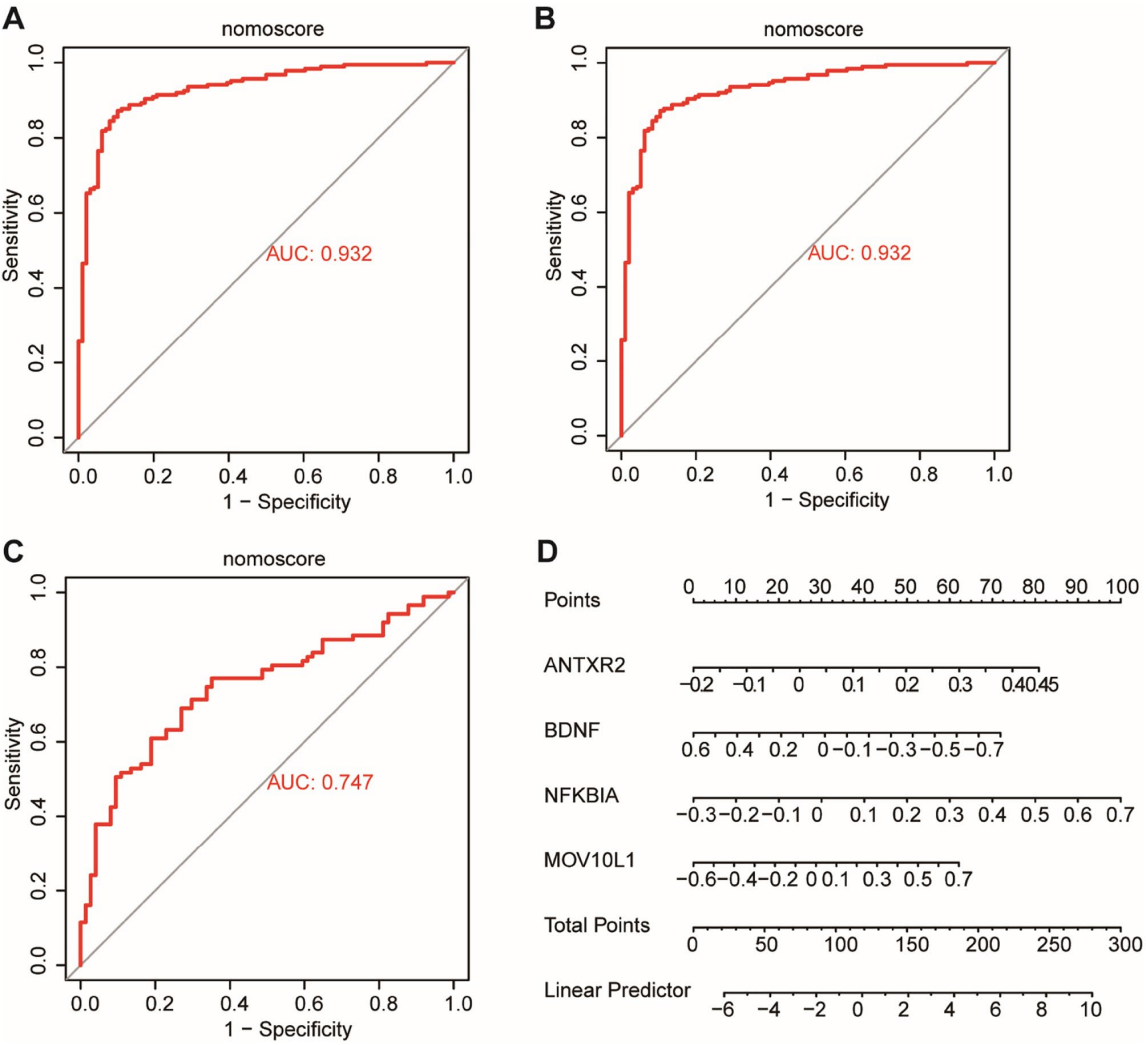
ROC curve analysis and construction of the nomogram model. (**A**–**C**) In the ROC curve, X-axis means (1-specificity). Meanwhile, the Y-axis exhibited the sensitivity in the ROC curve. We can find that the curve (AUC) was 0.932 in the training data, 0.924 in the test data, and 0.747 in the external validation set. (**D**) And Nomogram model integrating the risk score was constructed. The ROC curve of each candidate gene (ANTXR2, BDNF, NFKBIA, MOV10L1) and nomogram show the significant AD with hypoxia diagnostic value.

The area under the ROC curve (AUC) of the training set, test set and external validation set was used to verify the reliability of the diagnostic model (Fig. 6A–C). The optimized diagnostic model was constructed by the summation of “Exp ANTXR2 × 7.70567319783991 + ExpBDNF × -3.17991029321349 + ExpMOV10L1 × 2.95983099906647 + Exp NFKBIA × 6.19322860474308”. The AUC was 0.932 in the training dataset, 0.924 in the test dataset, and 0.747 in the external validation dataset. The AUC of all hub genes was greater than 0.7. A risk score system was constructed and assessed. The results of the nomogram analysis were shown in (Fig. 6D).

### Validation of hub genes

**Figure 7:**
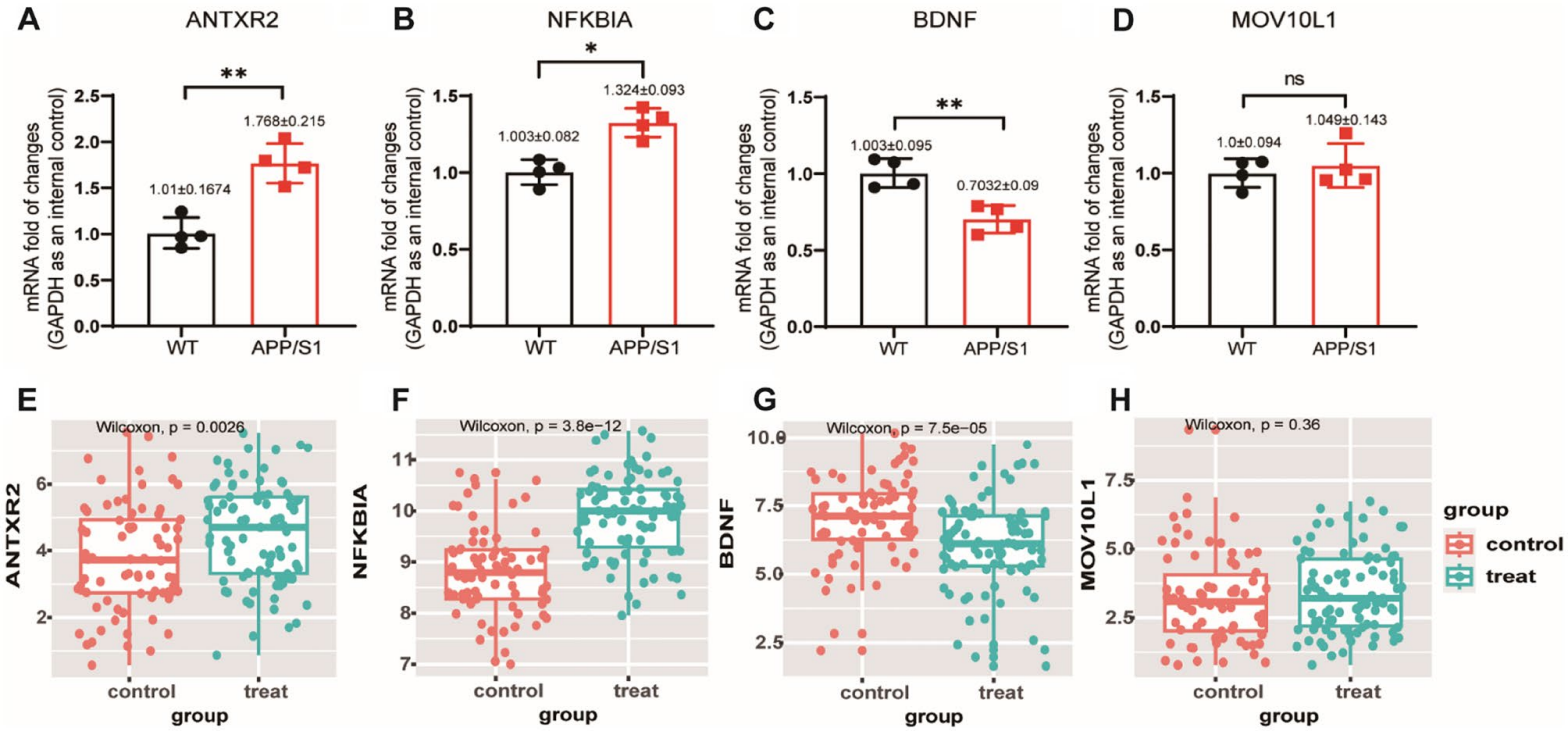
Validation of the four hub genes’ expression in APP/PS1 and wild-type mice by qRT-PCR and external dataset. (**A**–**D**) Validation the expression of hub genes. qRT-PCR results for mRNA levels of the hub genes NFKBIA, ANTXR2, BDNF and MOV10L1. As shown in the figure, ANTXR2 was significantly up-regulated in AD hippocampus (1.768 ± 0.215, P < 0.01), while NFKBIA was significantly up-regulated in AD hippocampus (1.324 ± 0.093, P < 0.05). In AD hippocampus (0.7032 + 0.09, P < 0.01). However, the difference of MOV10L1 gene between the two groups could not be determined because of the expression. Data are shown as mean ± SD. (*p < 0.05, **p < 0.01, ns, no significance). (**E**–**H**) Box plots were used to show the results of single gene difference analysis of NFKBIA, ANTXR2, BDNF, and MOV10L1 between AD and normal groups in the external validation set.

To verify the accuracy of bioinformatics analysis, qRT-PCR was performed on the hippocampus of four 6-month-old APP/PS1 mice and four wild-type mice. The expression of the four hub genes was measured (Fig. 7A–D). qRT-PCR results showed that, compared with the control group, the mRNA expression levels of NFKBIA and ANTXR2 in the hippocampus of mice in AD group was significantly higher (p < 0.05) than that in the Control, while mRNA expression level of BDNF in AD group was significantly lower (p < 0.05). Because the expression of MOV10L1 gene is low in brain tissue, no differences in mRNA expression levels in the control and AD groups were detected. The results of the external dataset suggested that NFKBIA, ANTXR2, and BDNF were differentially expressed between AD and normal, while MOV10L1 was not. (Fig. 7E–H).

## Discussion

With the rapid aging of the population worldwide, neurological dysfunction caused by AD has seriously affected the elderly's life quality^23^. Despite many efforts in developing therapeutic drugs, few effective measures, including donepezil, memantine, and bapineuzumab, can reverse the progression of the disease. Nevertheless, these drugs were mainly used to delay the progression of the disease in a limited way. More pieces of evidence link the pathophysiology of AD to hypoxia, which would significantly affect neuronal and brain performance^24^. While recent studies have revealed cerebral blood flow deficits in the initiation of cognitive decline, specific categories of dementia still need to be distinguished, such as AD and vascular cognitive impairment (VCI)^10^. The causes of VCI were mainly hemodynamic changes and the decrease in nerve cell activity^25^.

Chronic hypoxia is a significant environmental factor in the pathogenesis of neurodegeneration diseases and researchers have extensively demonstrated its multifaceted impacts on pathogenesis. For instance, studies have shown that hypoxia can induce oxidative stress and disrupt cellular energy metabolism, leading to neuronal damage and dysfunction^26^. Additionally, hypoxia has been found to promote the accumulation of misfolded proteins, such as beta-amyloid (Aβ) and tau, which are hallmark features of diseases like AD^27,28^. Moreover, hypoxia can trigger chronic inflammation and impair neurovascular functions, further exacerbating neurodegenerative processes^29^. These diverse impacts underscore the intricate relationship between hypoxia and the development of neurodegeneration, highlighting the need for targeted therapeutic strategies to mitigate its detrimental effects. However, the exact mechanism of AD remains unclear. The correlation between AD and hypoxia requires further explanation^30^. In recent years, given the rapid development of gene sequencing technology, bioinformatics research has contributed to understanding pathogenesis and biomarkers for the detection and treatment of AD. In this study, we have performed a transcriptomics study to elaborate on the underlying biological mechanisms between AD and hypoxia at the genetic level, which is conducive to promoting the accuracy of diagnosis in AD patients.

This study used a bioinformatic approach with machine learning to identify genes related to AD and hypoxia. A four-gene diagnostic model including ANTXR2, BDNF, NFKBIA, and MOV10L1 was constructed. This diagnostic model can be used to evaluate patients with hypoxia-associated AD and provide diagnostic biomarkers and therapeutic targets for AD patients. Then, we tested on APP/PS1 mice and wild-type mice with cognitive impairment phenotypes to verify the expression of hippocampal genes by qRT-PCR and to evaluate the reliability and validity of bioinformatics analysis.

ANTXR2 encodes a transmembrane protein called capillary morphogenesis protein 2(CMG2), plays important roles in endothelial proliferation, angiogenesis, cell adhesion and migration^31,32^. Additionally, CMG2 has been implicated in the modulation of immune responses^33^. Mutations in the ANTXR2 gene have been linked to several genetic disorders, including infantile systemic hyalinosis (ISH) and juvenile hyaline fibromatosis (JHF)^34^. It has been reported that the expression of ANTXR2 is related to nociceptive sensory neurons^35^. This gene is crucial to standard physiological processes. Some studies demonstrate that CMG2 regulates endosomal and lysosomal function as a collagen VI receptor^36^. Furthermore, ANTXR2 correlates with the low-density lipoprotein receptor-associated Protein 6 (LRP6) gene, which is a coreceptor for Wnt signaling. Late-onset AD might be involved in common LRP6 variants^37^. In addition, ANTXR2 is associated with changes in brain functional connectivity^38^. Through the function of HIF-1α, hypoxia can decrease EZH2 expression and increase ANTXR2 expression^39^. In the experimental verification, the aberrant expression of ANTXR2 is consistent with the trend from statistical analysis.

BDNF encodes a protein called brain-derived neurotrophic factor, which belongs to the family of neurotrophins. It can prevent neurodegeneration and promote neural regeneration^40^. BDNF is mainly expressed in the cerebral cortex and hippocampus, critical for memory, learning, and cognitive function^41^. Alterations in the BDNF gene have been associated with several neurological and psychiatric disorders. For example, certain genetic variations, such as single nucleotide polymorphisms (SNPs), in the BDNF gene have been linked to an increased risk of developing conditions like Alzheimer's disease, depression, schizophrenia, and bipolar disorder^42,43^. These variations may impact BDNF expression or protein function, leading to disrupted neurodevelopment or impaired synaptic plasticity. Influenced by the BDNF locus in chromosome 11, the circulating BDNF level participates in the severity of AD^44^. However, some researchers believe that low peripheral BDNF expression leads to AD^41^, while other studies demonstrated high peripheral BDNF expression in AD patients compared to controls^45^. These two conflicting conclusions deserve further study. Current studies have shown that the increased expression and content of BDNF in an anoxic environment can remodel microvessels and delay the progression of brain injury after hypoxia^46^. The complex regulation and function of BDNF are essential for advancing our knowledge of brain health and developing potential therapeutic interventions.

NFKBIA can inhibit the function of nuclear factor-kappa B (NF-κB) as a direct target of NFIL3^47^. Studies have shown that the activation of NF-κB , which is a transcription factor involved in various cellular processes, plays a role in the pathogenesis of AD. NF-κB can be activated by inflammatory cytokines to control the development of inflammatory responses and is involved in anti-apoptotic transcription, glial activation, and other biological activities^48^. NF-κB has both neurotoxic and neuroprotective effects. It can protect by depositing Aβ and thus inducing dementia under pathological conditions. In addition, itt can protect nerve function from oxidative stress under physiological conditions^49^. NFKBIA has been found to interact with HIF-1α. Studies suggest that inhibiting NF-κB can potentially decrease the expression of HIF-1α under hypoxic conditions^50^. In the case of hypoxia, the overactivation of inflammation-related pathways would worsen brain damage.

Both MOV10L1 and MOV10 are RNA helicase homologs involved in various biological processes, such as resistance to RNA viruses, regulation of neuronal function, influence on reproductive function, and brain development^51^. Mov10L1 is identified as a gene specifically required for regulating the reproductive system and is essential for producing piRNAs^52,53^. Researchers have found that the expression of piRNA in animal models may exhibit significant changes under hypoxic conditions^54^. Furthermore, PIWI proteins can bind to PIWI-interacting RNAs (piRNAs), which may regulate retrotransposons in the central nervous system^55^. It has been reported that improper retrotransposons regulation may be associated with degenerative neuropathy^56^. We speculate that MOV10L1 was related to the pathogenesis of AD, but the exact content still needs further study. The results were verified by qRT-PCR in the brain tissue of APP/PS1 and wild-type mice. Through repeated experiments, it was found that the expression of MOV10L1 gene in mouse brain tissue could not be detected. Since we couldn’t obtain brain tissues of AD patients for qRT-PCR experiments at present, we used the external dataset to verify the expression of MOV10L1. Then we found that there was no difference between AD patients and normal people, so its predictive value still remained to be evaluated. The results from the ROC in the training dataset, test dataset, and external validation dataset suggested that micro-environmental hypoxia plays a vital role in AD. The single-gene GSEA analysis also indicated that the oxidative phosphorylation pathway was inseparable from AD and hypoxia.

Recently, a growing amount of evidence supports the critical role of genes in disease diagnostic screening. Early identification and intervention of AD would shorten the treatment time and improve the therapeutic effect. Based on various research, hypoxia has been shown to play an essential role in the development of AD. However, the diagnostic link between hypoxia and AD has yet to be thoroughly studied. Through bioinformatics analysis, we aimed to screen characteristic genes for their diagnostic values for AD patients. After further differentiating the DEGs into modules, we found that these hub genes were mainly involved in the AD pathway, reactive oxygen species, autophagy, cAMP signaling pathway and other processes, all suggesting that AD is closely related to hypoxia. Furthermore, related genes screened by machine learning and the nomogram model showed great predictive ability and clinical usefulness. The ROC verification followed by the qRT-PCR verification significantly validates the accuracy of the results. The significant correlation between hypoxia related genes (NFKBIA, ANTXR2, and BDNF) and AD suggests that these signature genes have the predictive value for the diagnosis of AD. The present study provided novel insights from the genetic perspective. Genetic biomarkers that demonstrated predictive values for AD patients were also identified and validated in fundamental experiment, which promotes the prediction accuracy for diagnosis of AD.

This study also has some limitations. First, the sample of laboratory proof needs to have the same sample source with transcriptome database. However, it is difficult to obtain human brain samples, so we used mice to verify the analysis data. Second, the expression of genes is related to tissue sites, and the results are only for preliminary verification. Third, the sample size for external validation needs to be increased. To assess the diagnostic accuracy of the model, large-scale samples are urgently required.

In summary, this study is the first innovative attempt to study AD and hypoxia-related genes using bioinformatics. We used multiple analysis methods to identify three signature genes (NFKBIA, ANTXR2, BDNF), which were closely related to the pathogenesis of AD. Through data verification and fundamental experiment study, the expression of NFKBIA, ANTXR2 and BDNF was detected to be significantly regulated in AD patients. This discovery would provide a valuable reference for future clinical practice.

## Methods

### Source of gene expression microarray data and hypoxia-related genes

Two microarray datasets GSE33000^57^, and GSE5281^58^ were obtained from the GEO website database. We used the sample data in GSE33000, including expression profiles of postmortem prefrontal cortex samples from 467 AD patients and nondemented control individuals. This is the information available in publicly available databases. The brain tissue data was obtained from the Harvard Brain Tissue Resource Center (HBTRC). The majority of the HBTRC samples were of Caucasian ancestry. Among the AD patients, 135 were male and 175 were female, with average ages of 80.6 (SD = 9.0) years. The normal control group consisted of 123 males and 34 females, with an average age of 63.5 (SD = 9.9) years. For validation set information GSE5281, which includes the AD patients, 37 were male and 50 were female, with an average age of 79.8 years. The control group consisted of 21 males and 53 females with a mean age of 79.5 years. Then, we downloaded a set of hypoxia genes^59^ (n = 235) from the Molecular Signatures Database (MSigDB) website (https://www.gsea-msigdb.org/gsea/msigdb/). This gene set has been commonly used for hypoxia-associated analyses. We used the R software to screen the AD genes associated with hypoxia. And the workflow of this study was shown in (Fig. S1).

### Analysis of differentially expressed genes and unsupervised clustering

In this study, we utilized the “limma" in R to screen out differentially expressed genes (DEGs) in the training dataset. Differential gene expression analysis can identify variance between normal and control, then we used P-value < 0.05, | (log2FC) |> 0.05 as its screening criteria. We will present the DEGs by heatmap and volcano plot. In this study, we utilized the k-means machine learning algorithm, available through the "ConsensusClusterPlus" R package, to perform clustering analysis. To identify samples associated with hypoxia in the AD group, we employed the proportion of ambiguous clustering (PAC) algorithm and the cumulative distribution function (CDF) curves to filter two distinct clusters that could be used to assess hypoxia circumstances. Then, we conducted a differential gene expression analysis in the "hypoxia-low" and "hypoxia-high" groups.

### Weighted gene co-expression network analysis to identify hub genes

We used the WGCNA package in R software to identify modules and genes related to hypoxia^60^. Firstly, we conducted the clustering analysis to classify DEGs for AD vs. Control groups, and hypoxia-high and hypoxia-low groups. To ensure that gene correlation is closely related to scale-free distribution, we used an algorithm that can select the optimal soft threshold. |Correlation coefficient|> 0.6 and |p-value|< 0.05 were set as the criteria for the next operation, and | GS |> 0.62 and |MM|> 0.82 as the criteria to determine the hub genes.

### Gene ontology and Kyoto encyclopedia of genes and genomes pathway enrichment analysis

According to WGCNA results, 205 genes were selected for KEGG and GO enrichment analysis. This analysis included exploring the biological process (BP), cellular component (CC), and molecular function (MF) of the hub genes. Additionally, we performed KEGG and GO enrichment analysis with the R package "clusterProfiler" and “enrichplot”.

### Genes filtered by multiple machine learning algorithms

Machine learning is suitable for identifying critical genes with biomarkers in Alzheimer's Disease (AD). We utilized the LASSO regression, SVM-RFE, random forest (R.F.) and XGBoost algorithms to carry out feature screening, select the most appropriate gene, and construct the prediction model. The "glmnet", " randomForest", "xgboost", and the "e1071" packages participated in the genes filtering process.

### Single gene-set enrichment analysis of marker gene

Predictive genes were identified with machine learning. The “clusterProfiler”, “enrichplot”, and “patchwork” packages in R software were used to perform the Gene Set Enrichment Analysis (GSEA). GSEA is a vital method for selecting critical paths and functional phenotypes. In the GSEA, CI, correlation coefficients, and other indices were calculated by R. The significant level was set at 0.05. We performed GSEA analysis on single gene-set to better reflect the function of each gene.

### Evaluate the predictive power of diagnostic models

The “caret” in R was used to randomly divide the samples in GSE33000 into training set (70%) and test set (30%). In internal and external data, the receiver operating curve (ROC) analysis in “pROC” package was used to calculate the area under the ROC curve (AUC) and to evaluate the diagnostic value of the critical genes. The “rms” R package was used to build the nomogram model for clinical AD diagnosis.

### Quantitative real-time PCR (qRT-PCR) and validation with an external dataset

Four C57 APP/PS1 mice and four C57 wild-type mice were used as experimental animals. Twenty-four weeks old male mice were sacrificed by cervical dislocation, and the hippocampus of the mice was dissected and stored at − 80 °C for later use. Total RNA was extracted from the hippocampus of 6-month-old APP/PS1 mice and wild-type mice using TRIzol^®^ reagent according to the manufacturer’s instructions. cDNA was synthesized from 1 μg total RNA using a Vazyme reverse transcription kit (Nanjing, China). Quantitative real-time PCR analysis was carried out with the SYBR Green Master Mix (Thermo Fisher Scientific, MA, USA). The expression level of the four genes related to hypoxia and AD was determined by the 2^−ΔΔCt^ method. *β-actin was used as a normalized control*. At the same times, the external validation set GSE5281 was used to analyze the differences in the expression of the four genes between the AD and normal groups.

### Supplementary Information

Supplementary Information.

Supplementary Figure S1.

## Data Availability

The datasets generated and analyzed during the current study are available in the GEO and MSigDB database, https://www.ncbi.nlm.nih.gov/geo/query/acc.cgi; https://www.gsea-msigdb.org/gsea/msigdb/index.jsp.
