# Supplementary material for: Identification and verification of genes associated with hypoxia microenvironment in Alzheimer’s disease
Source: Sci Rep. 2023 Sep 27;13:16252. doi: 10.1038/s41598-023-43595-9 (PMC10533856; doi:10.1038/s41598-023-43595-9)
Supplement: Supplementary file 1 — Supplementary Information. [file 41598_2023_43595_MOESM1_ESM.zip › row data/Test group raw figure/consensus.pdf]

consensus matrix legend

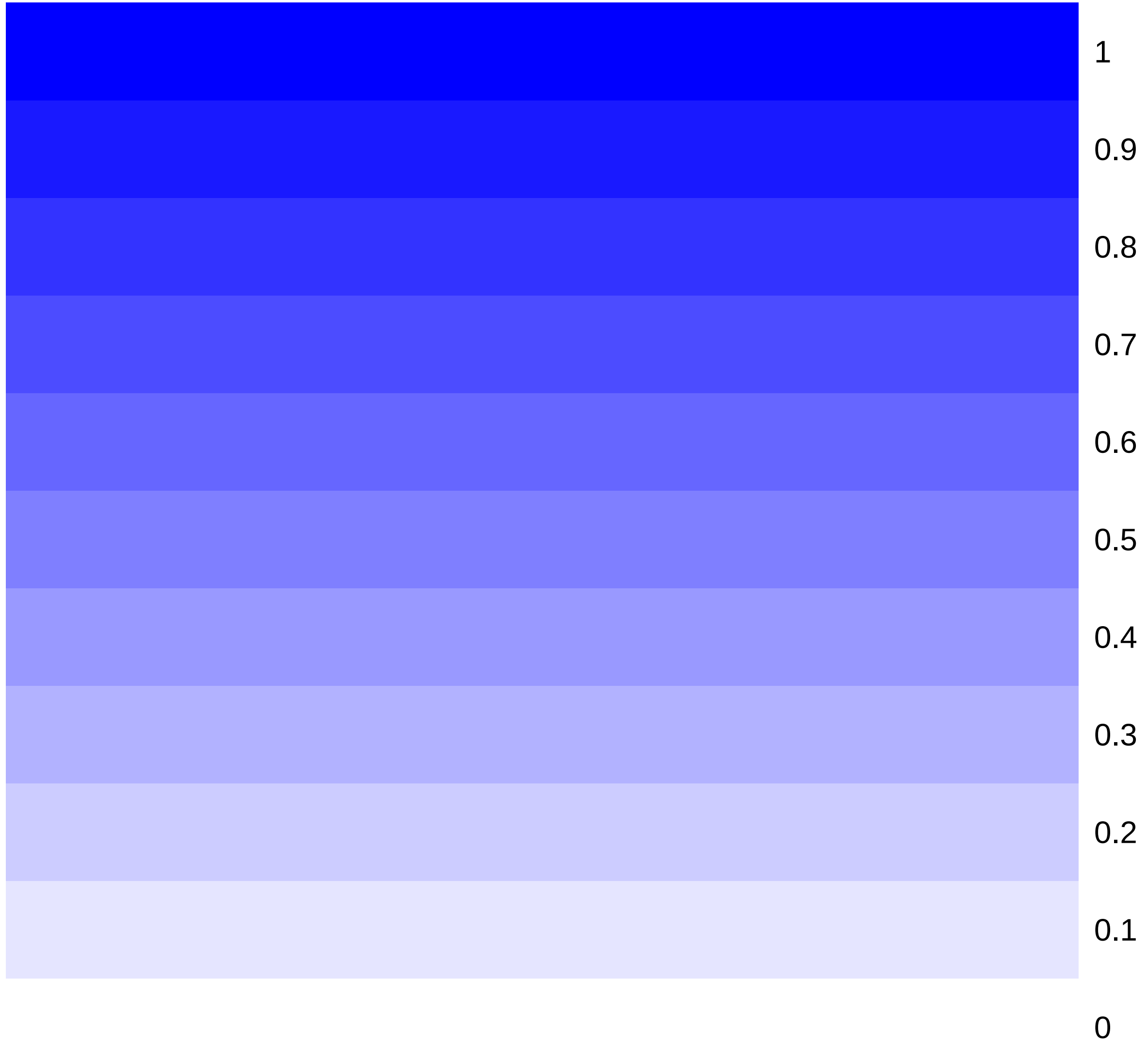

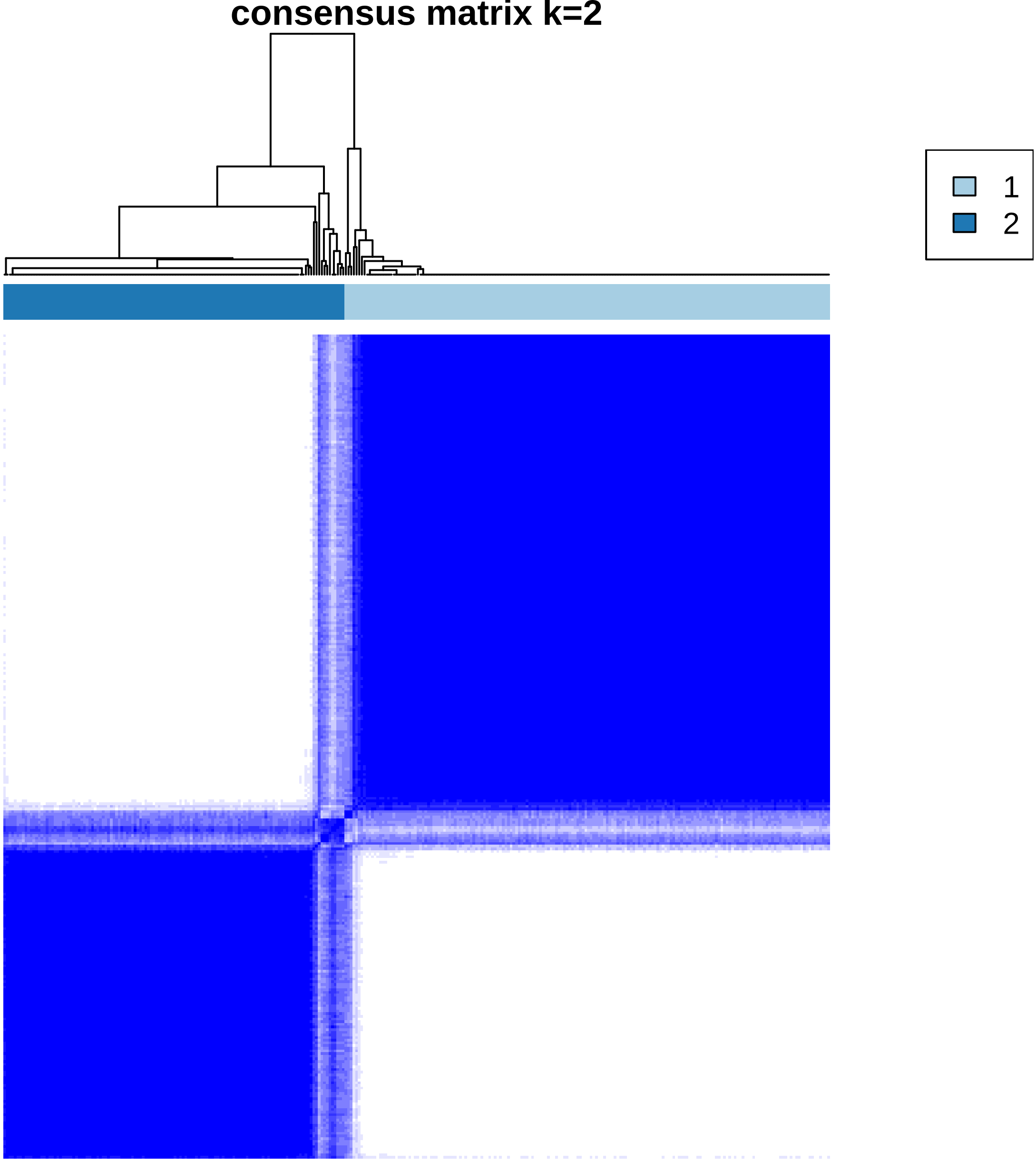
consensus matrix k=2
1
2

consensus matrix k=3

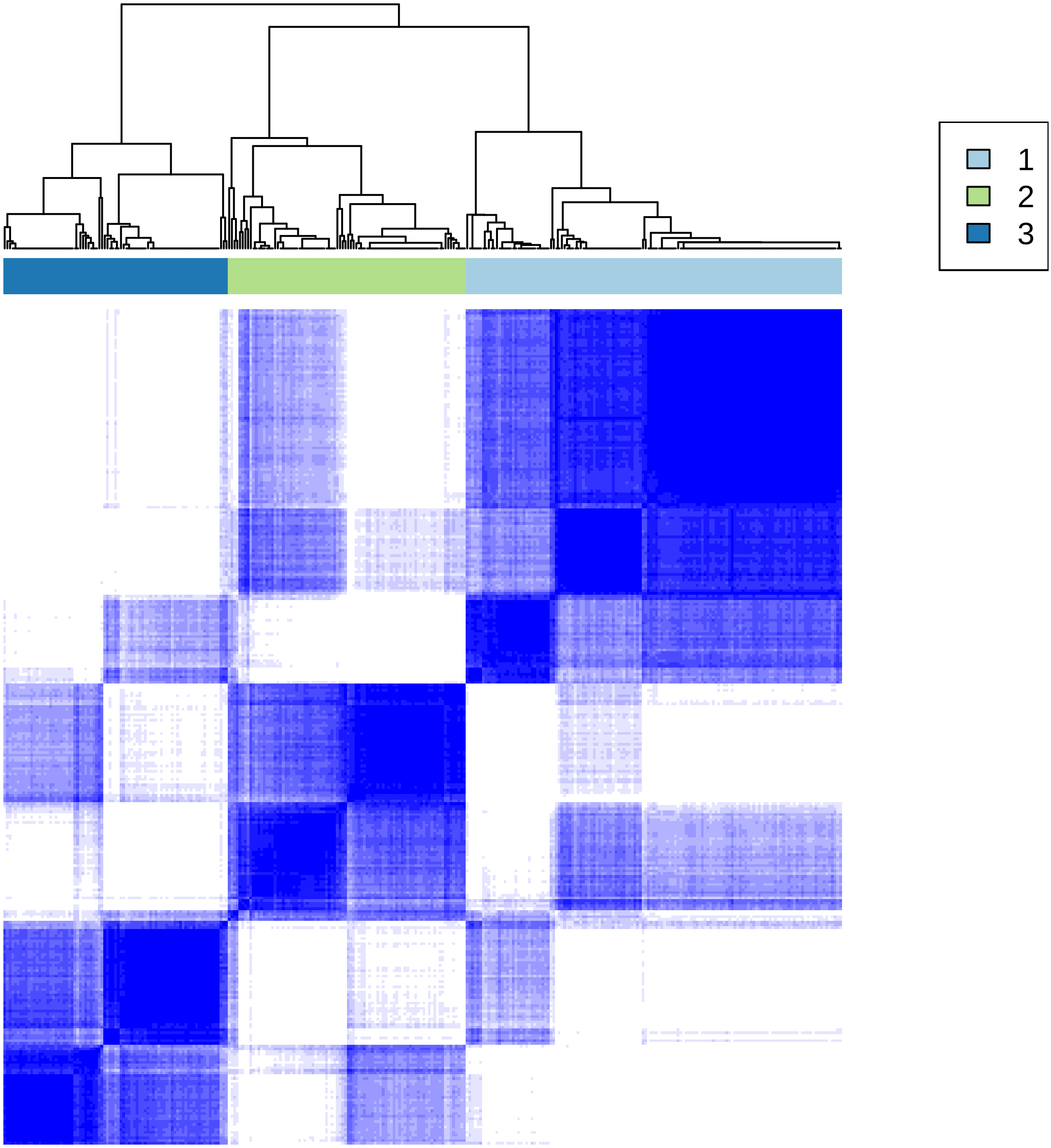

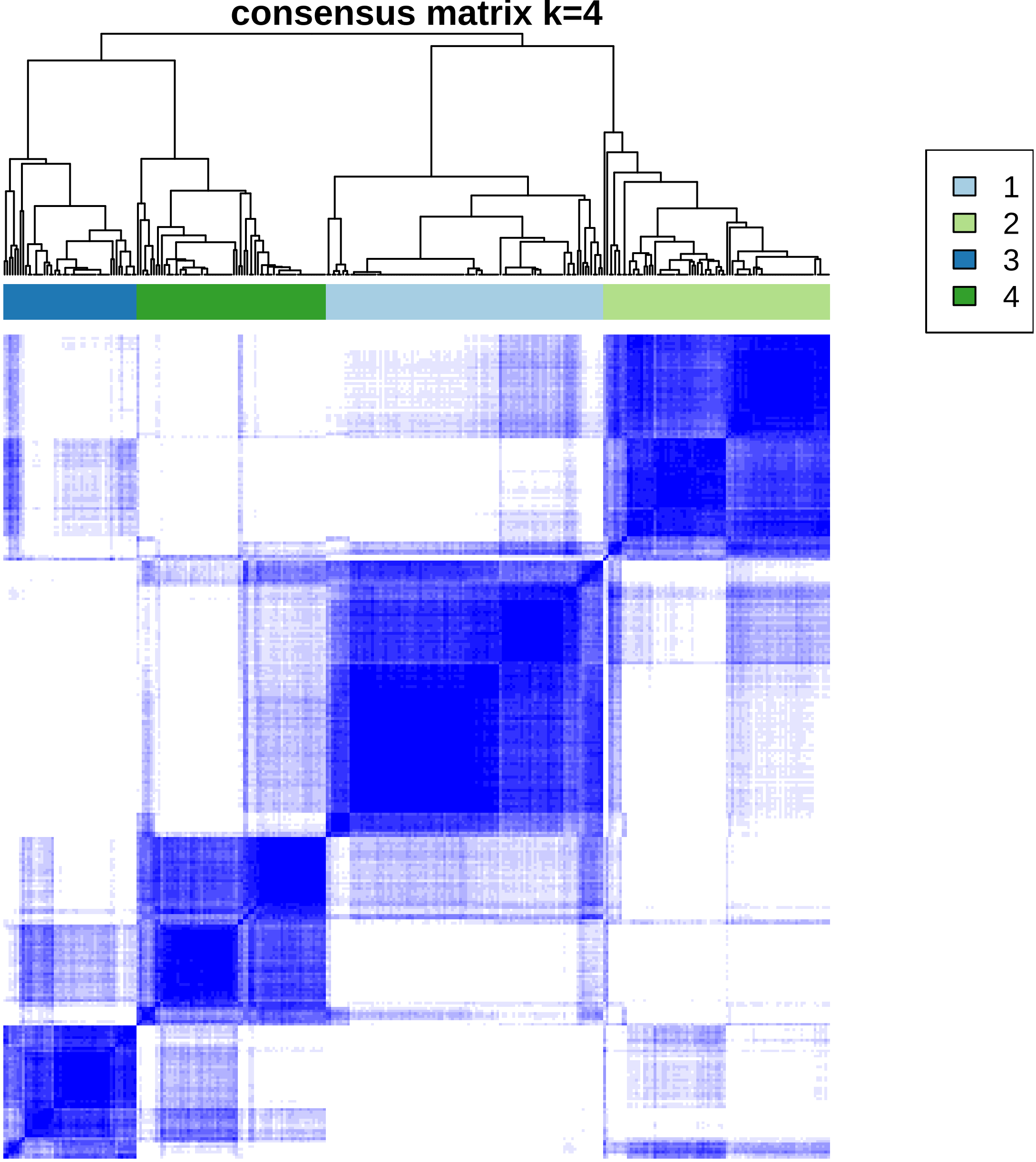
consensus matrix k=4
1
2
3
4

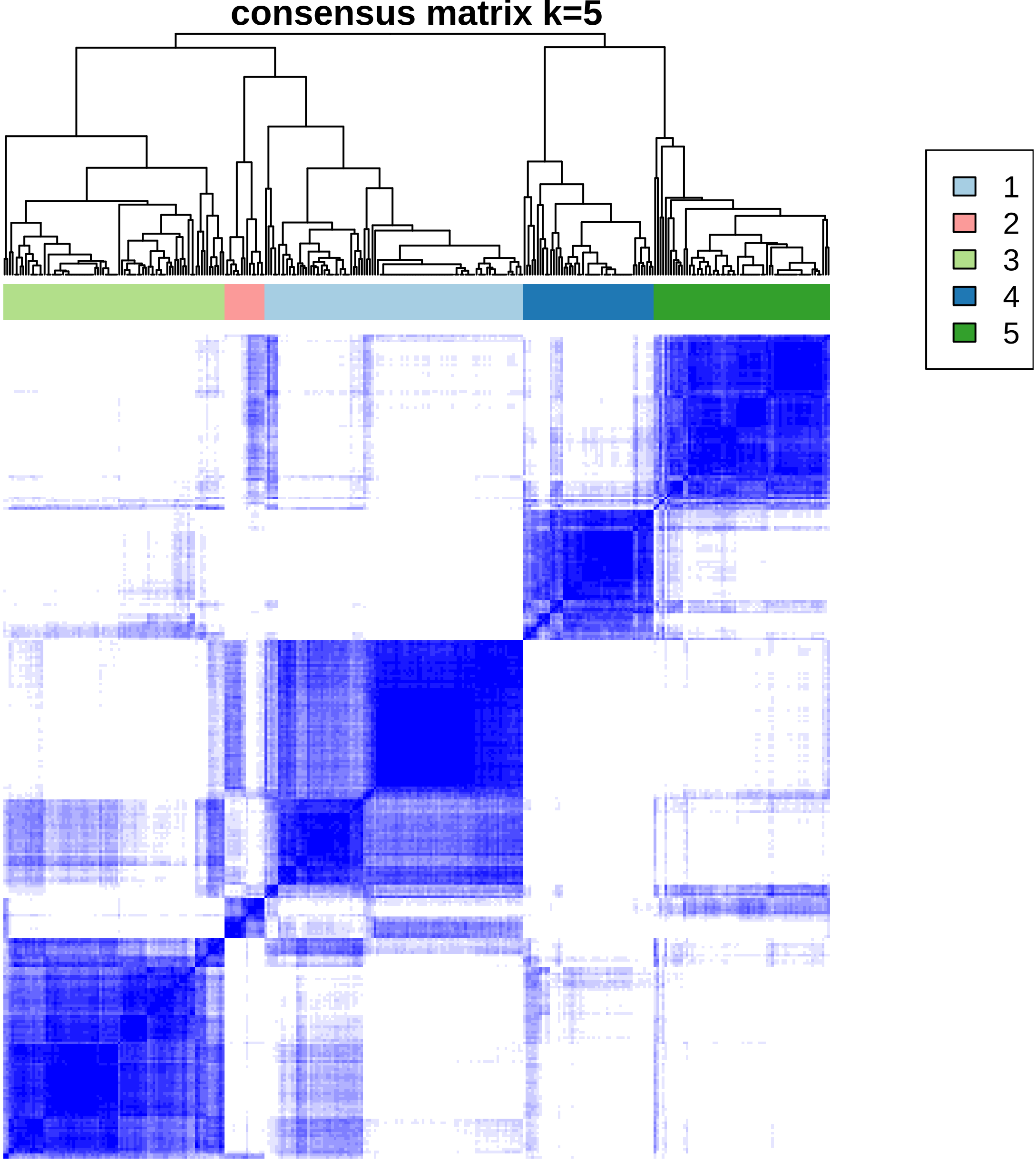
consensus matrix k=5
1
2
3
4
5

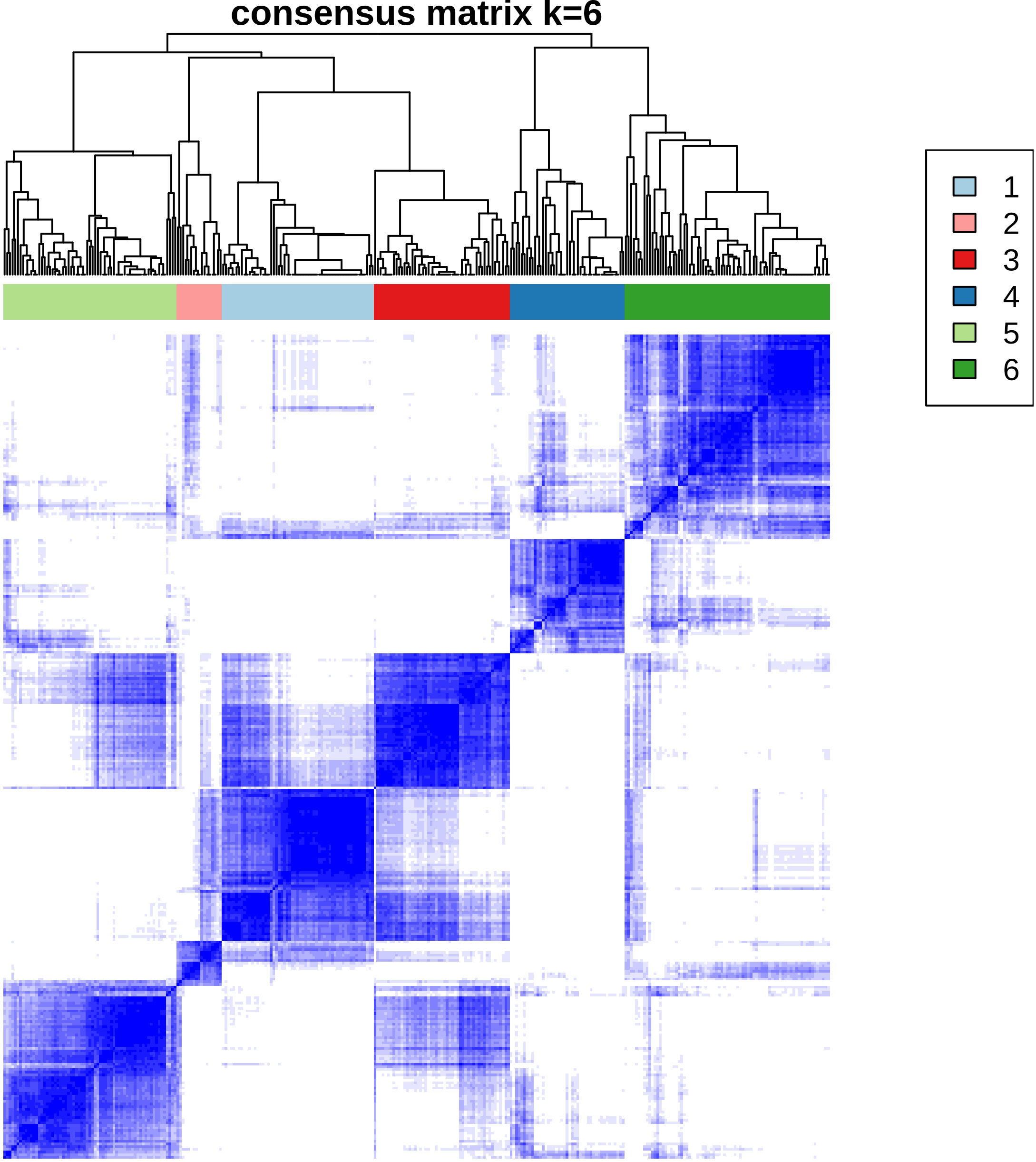
consensus matrix k=6
1
2
3
4
5
6

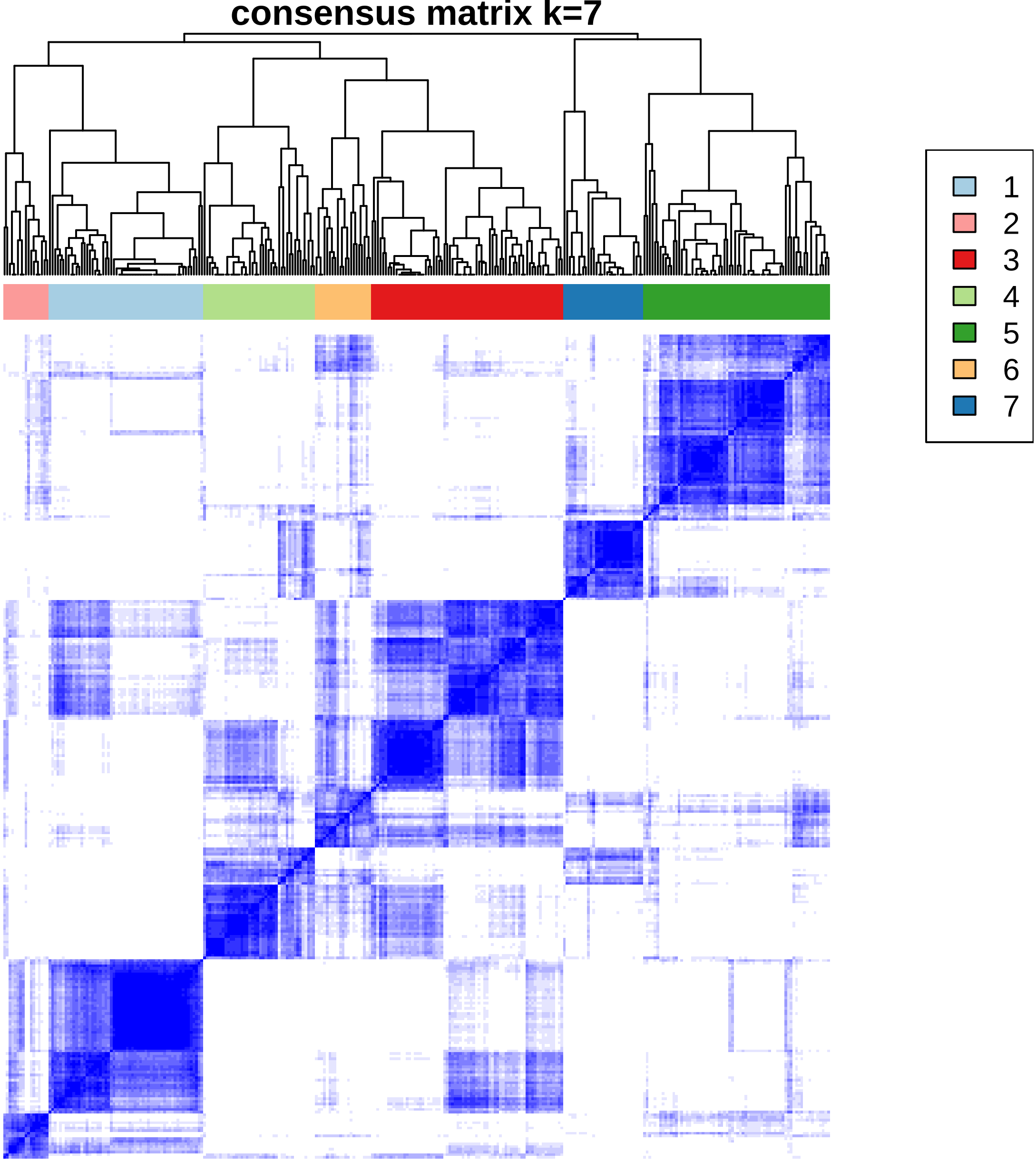
consensus matrix k=7
1
2
3
4
5
6
7

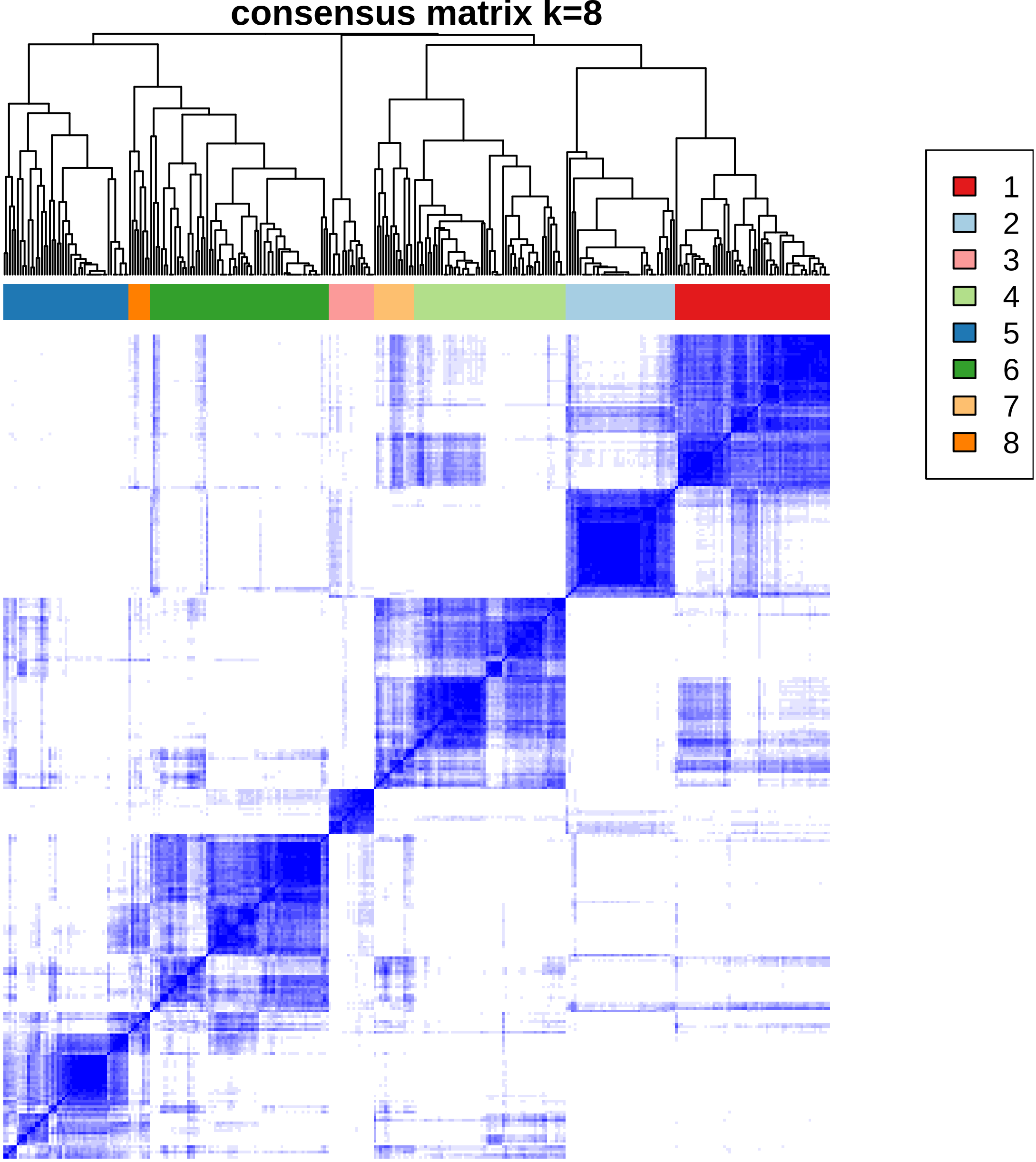
consensus matrix k=8
1
2
3
4
5
6
7
8

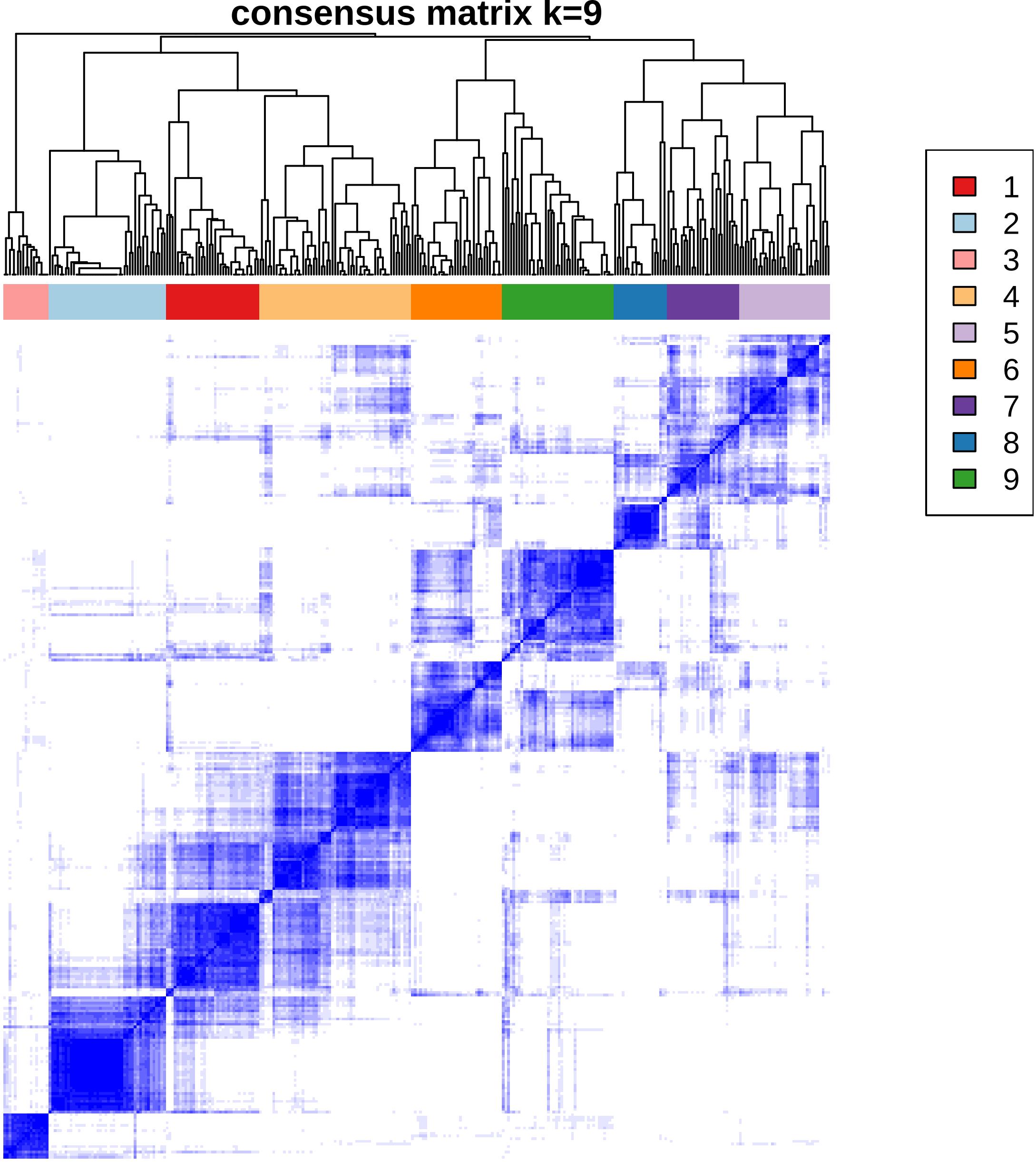

consensus matrix k=9
1
2
3
4
5
6
7
8
9

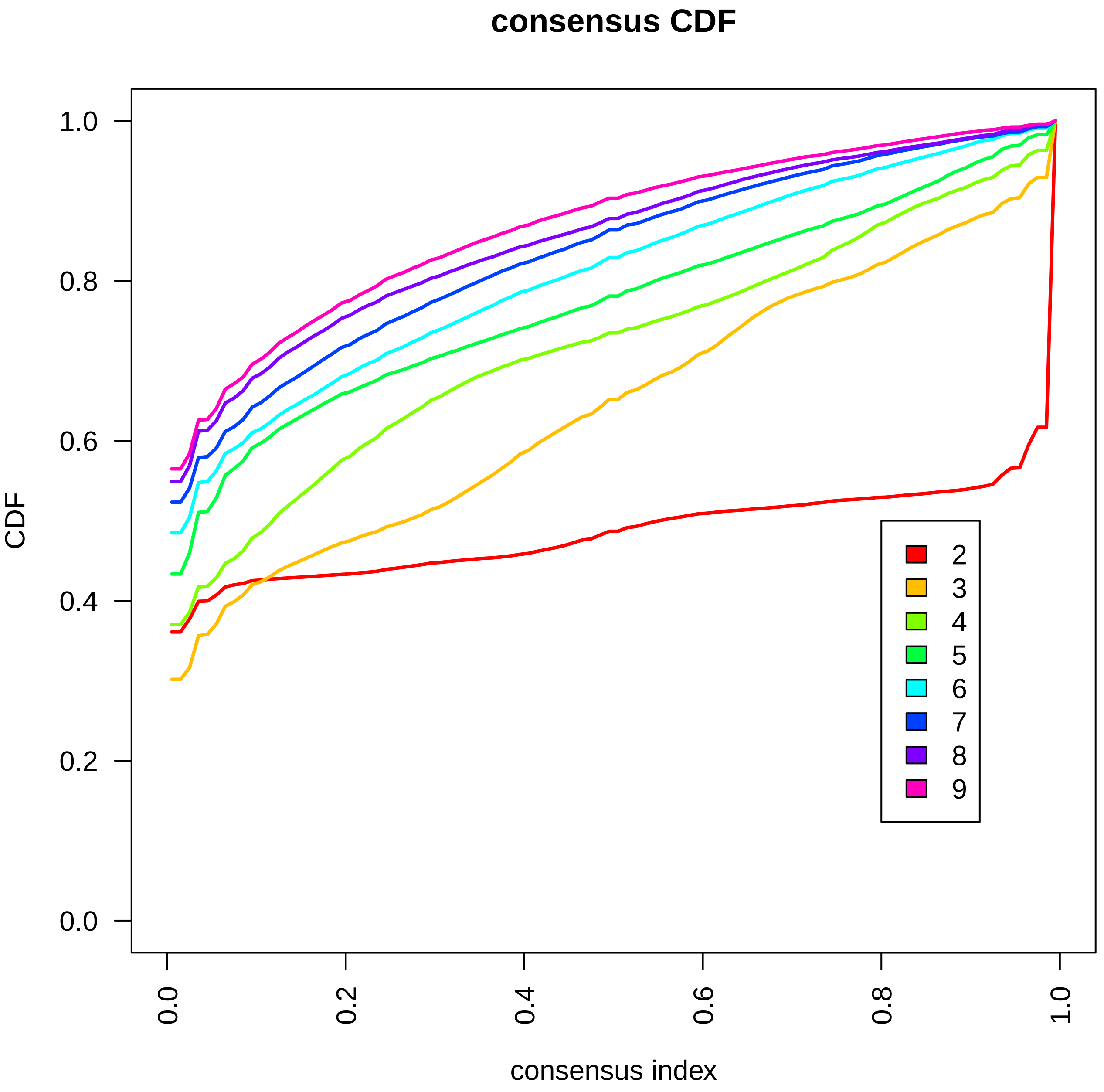
consensus CDF
CDF
consensus index
0.0
0.2
0.4
0.6
0.8
1.0
0.0
0.2
0.4
0.6
0.8
1.0
2
3
4
5
6
7
8
9

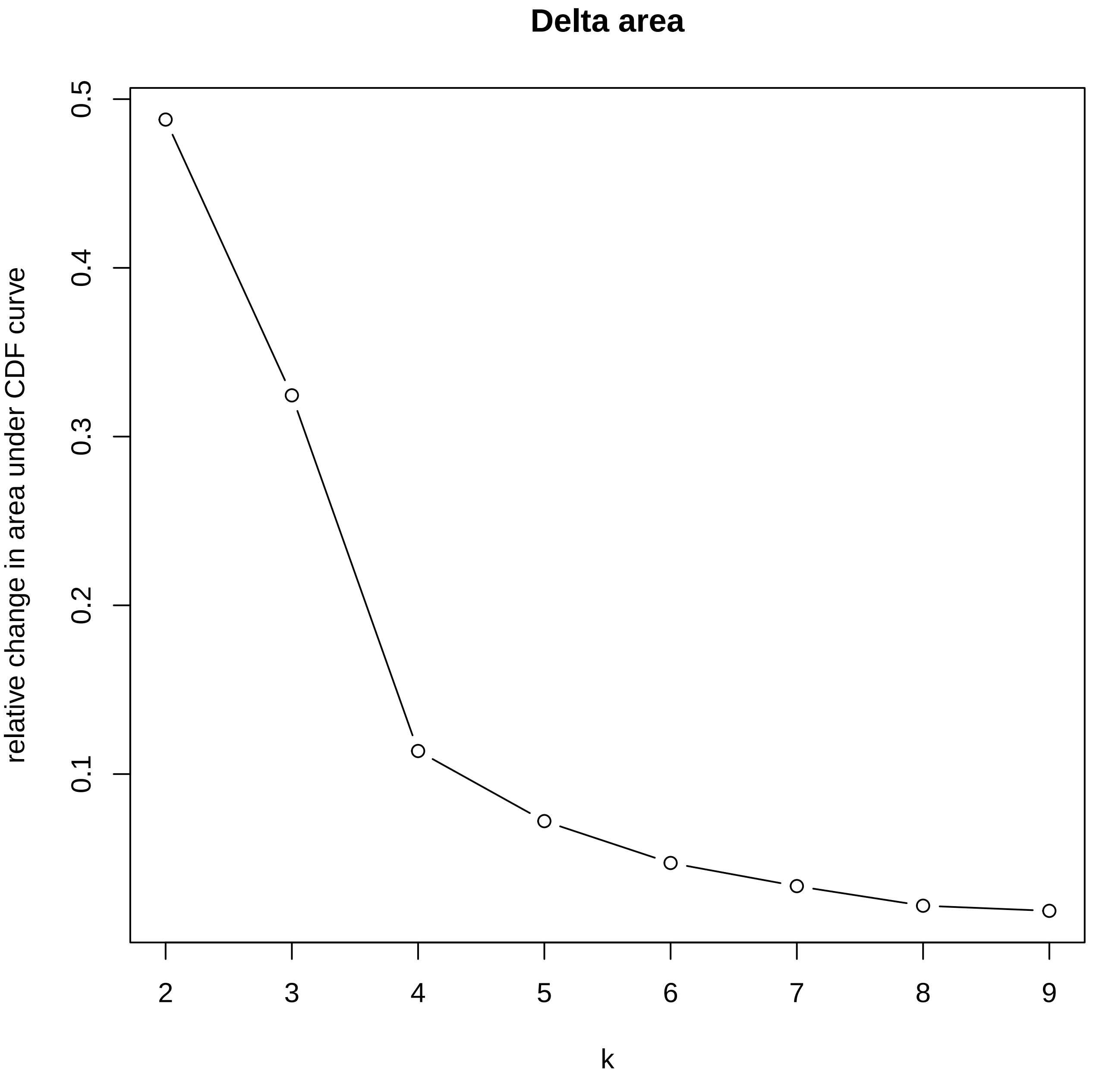
Delta area
relative change in area under CDF curve
0.1
0.2
0.3
0.4
0.5
2
3
4
5
6
7
8
9
k

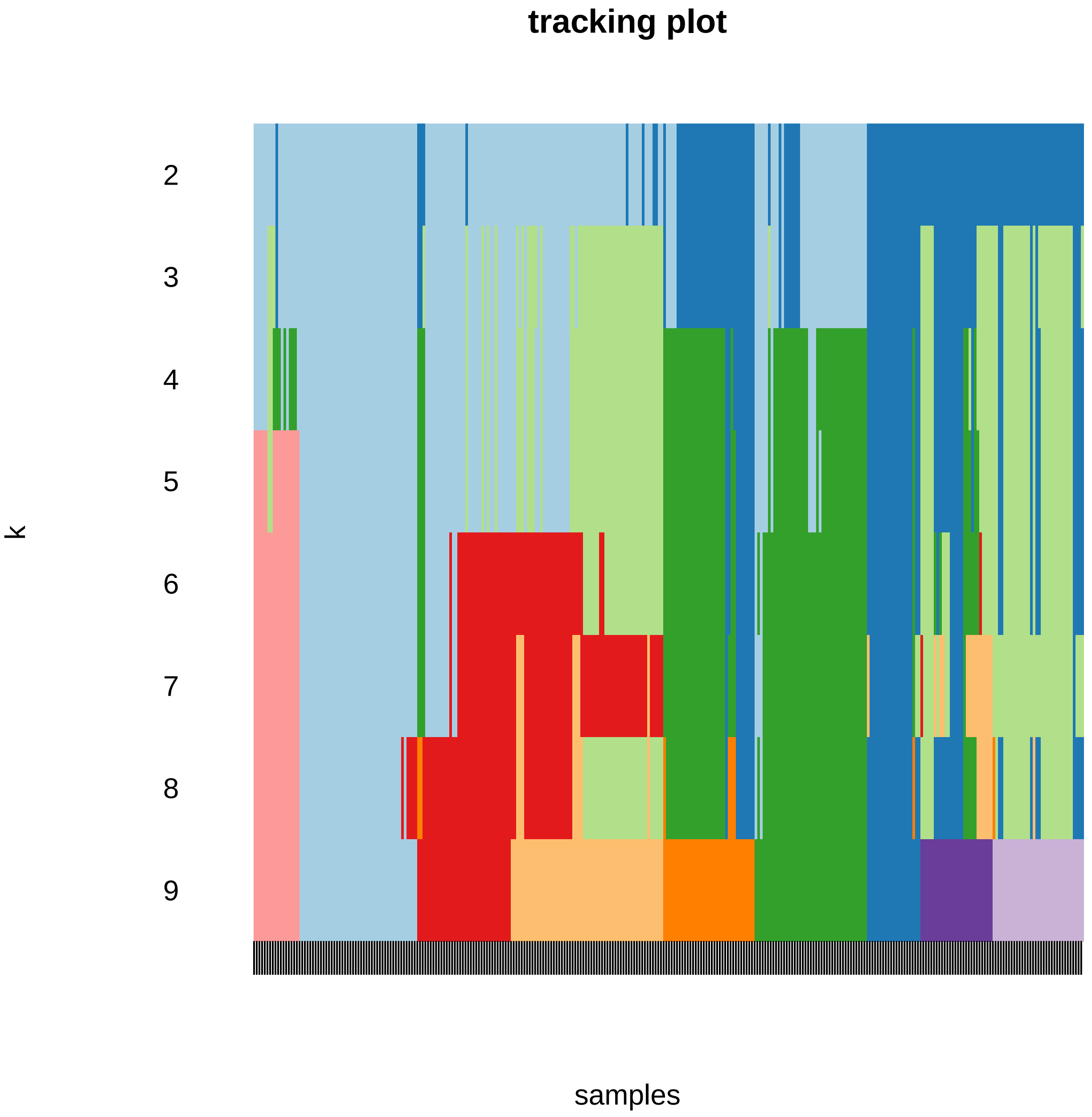
tracking plot
2
3
4
5
6
7
8
9
k
samples
