# Supplementary figures and images for: Identification and verification of genes associated with hypoxia microenvironment in Alzheimer’s disease
Source: Sci Rep. 2023 Sep 27;13:16252. doi: 10.1038/s41598-023-43595-9 (PMC10533856; doi:10.1038/s41598-023-43595-9)

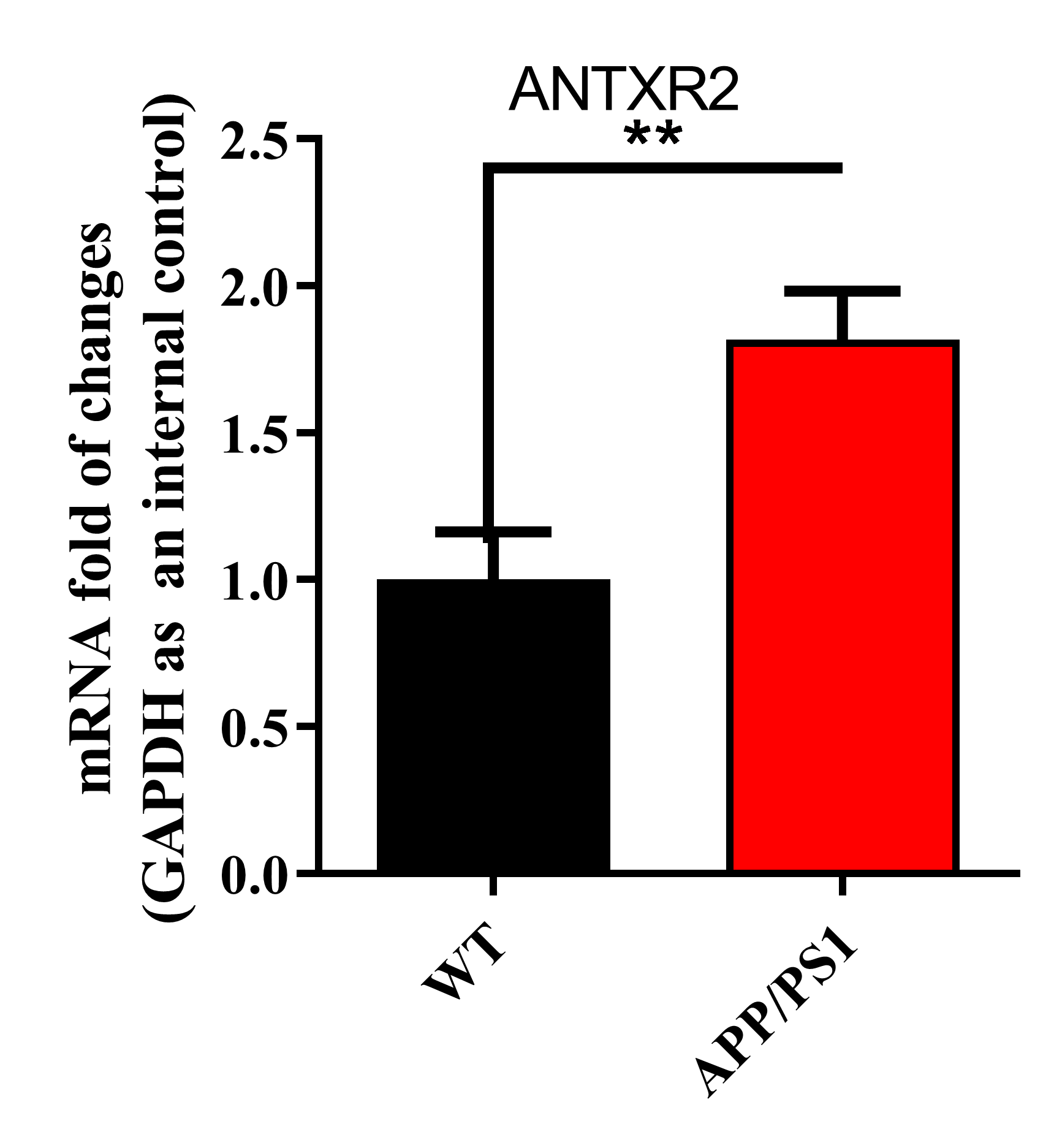

Supplement: Supplementary file 1 — Supplementary Information. [file 41598_2023_43595_MOESM1_ESM.zip › row data/qRT-PCR/ANTXR2.tif]

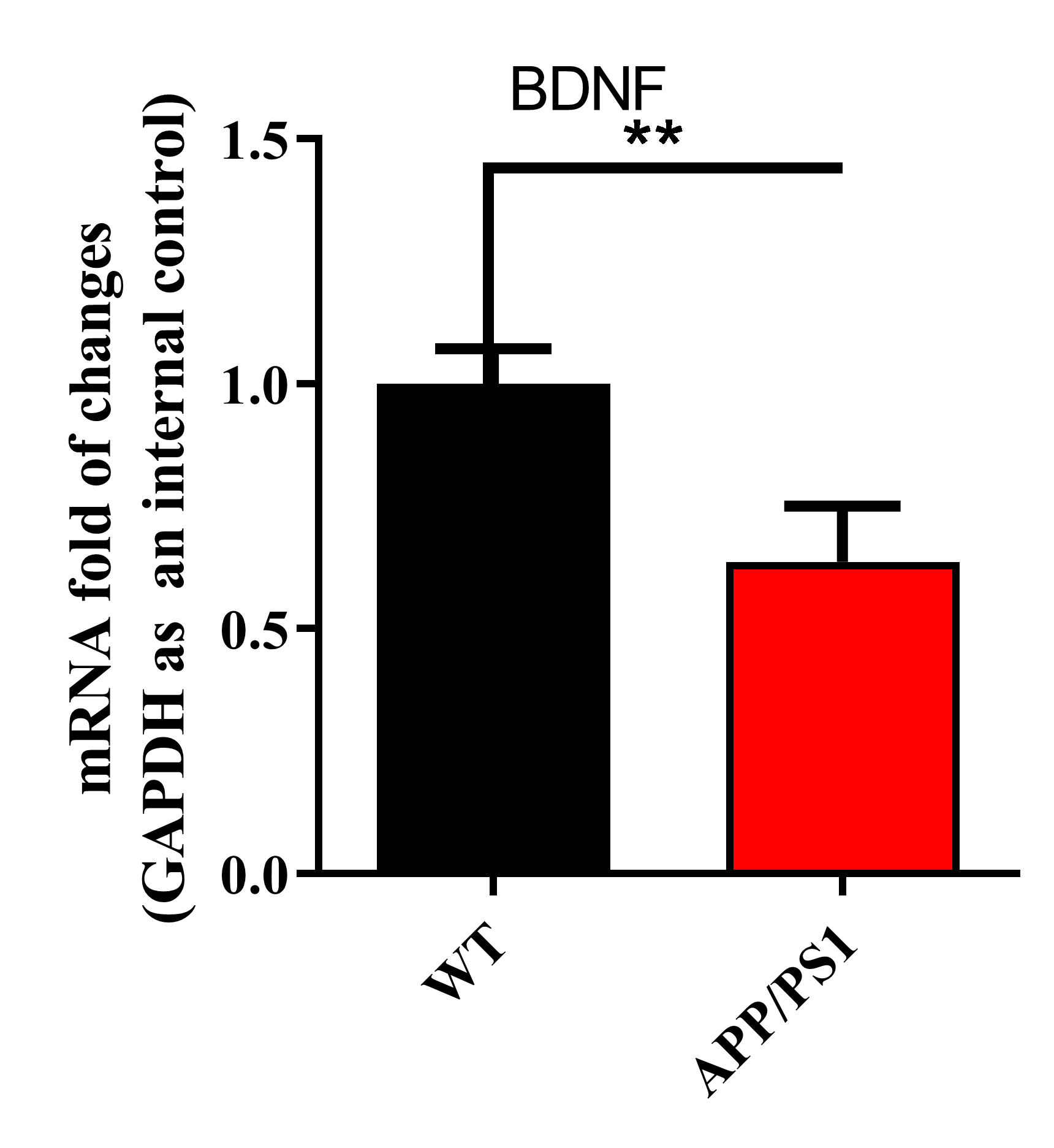

Supplement: Supplementary file 1 — Supplementary Information. [file 41598_2023_43595_MOESM1_ESM.zip › row data/qRT-PCR/BDNF.tif]

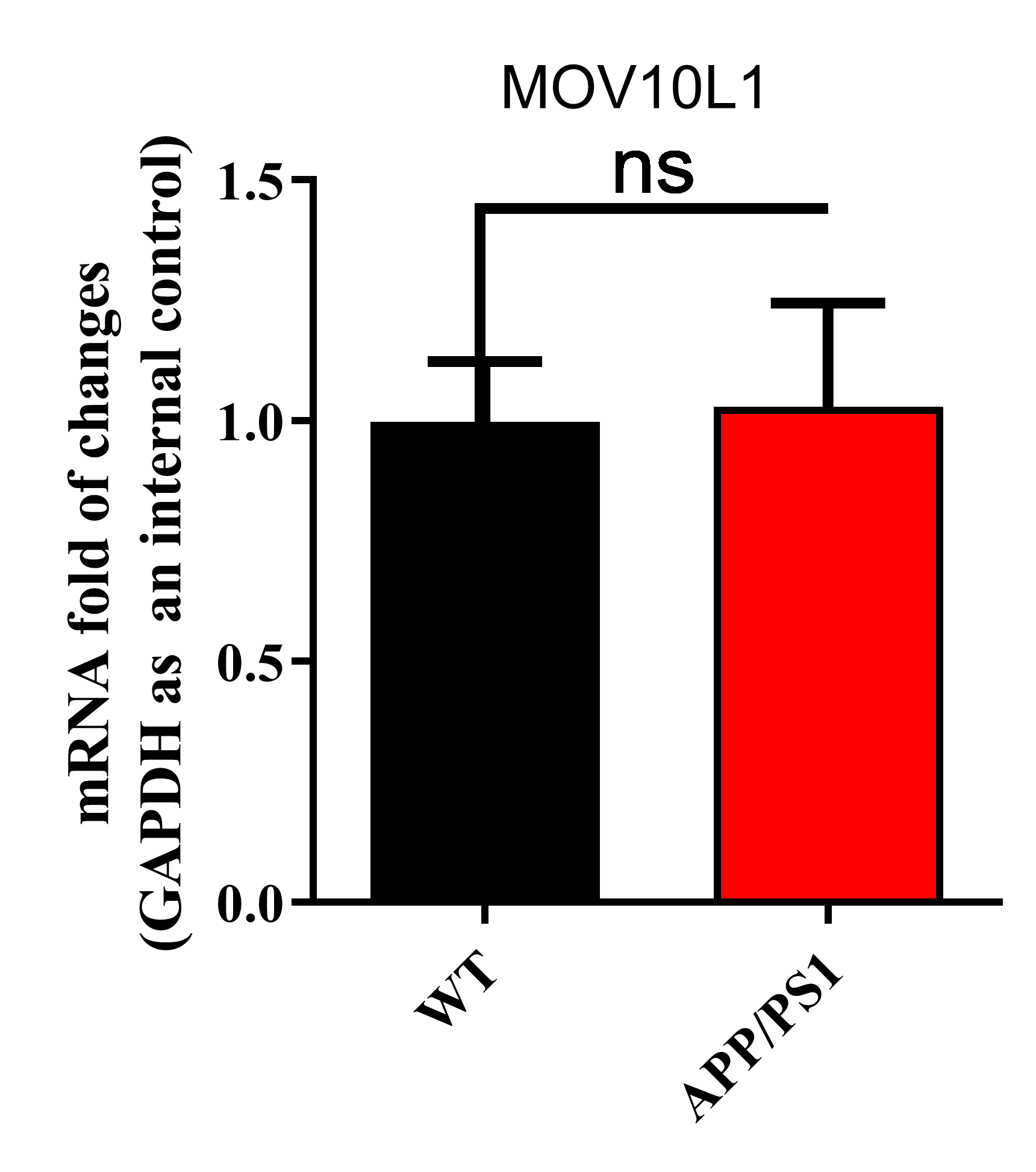

Supplement: Supplementary file 1 — Supplementary Information. [file 41598_2023_43595_MOESM1_ESM.zip › row data/qRT-PCR/MOV10L1(1).tif]

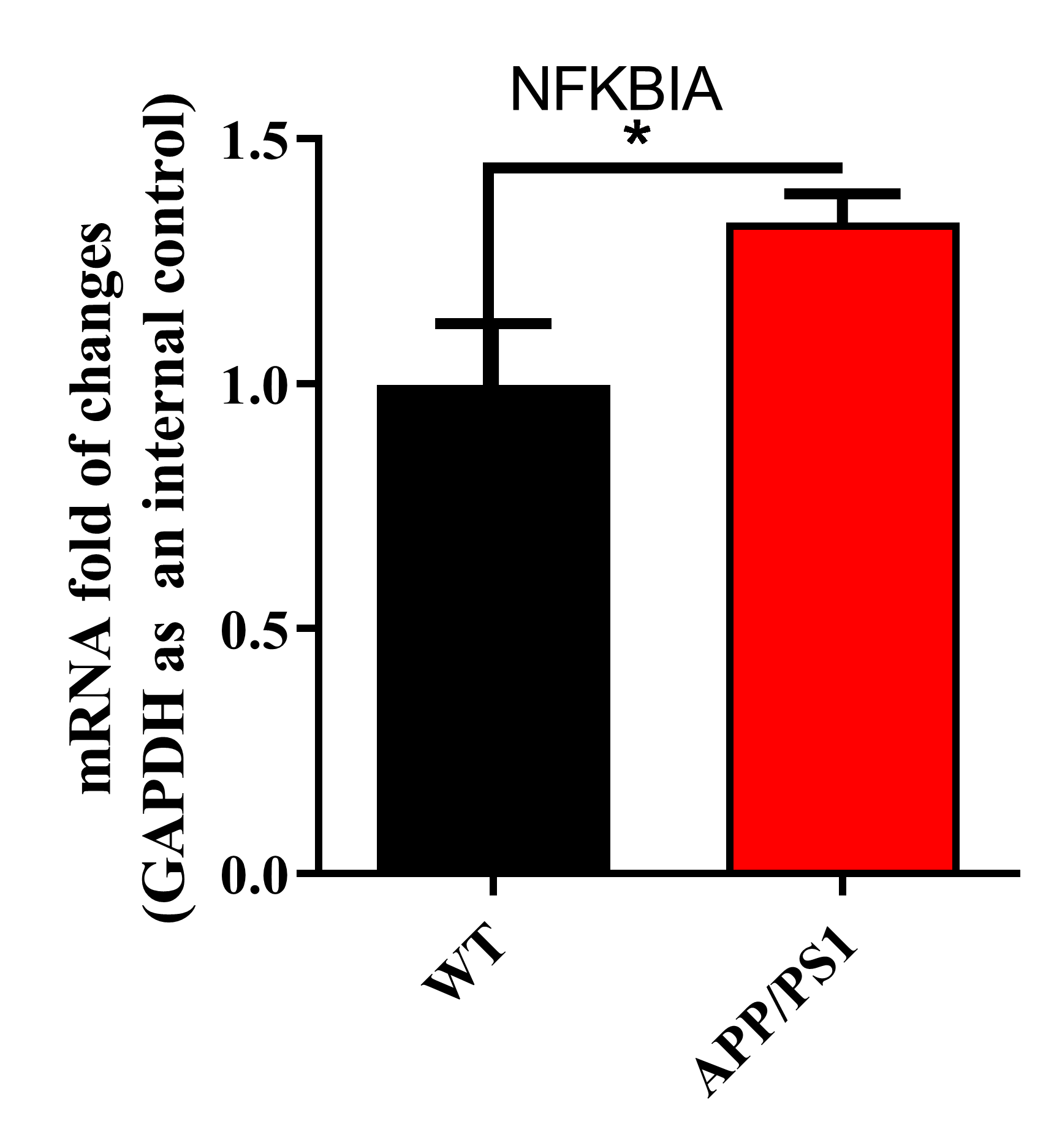

Supplement: Supplementary file 1 — Supplementary Information. [file 41598_2023_43595_MOESM1_ESM.zip › row data/qRT-PCR/NFKB1A.tif]

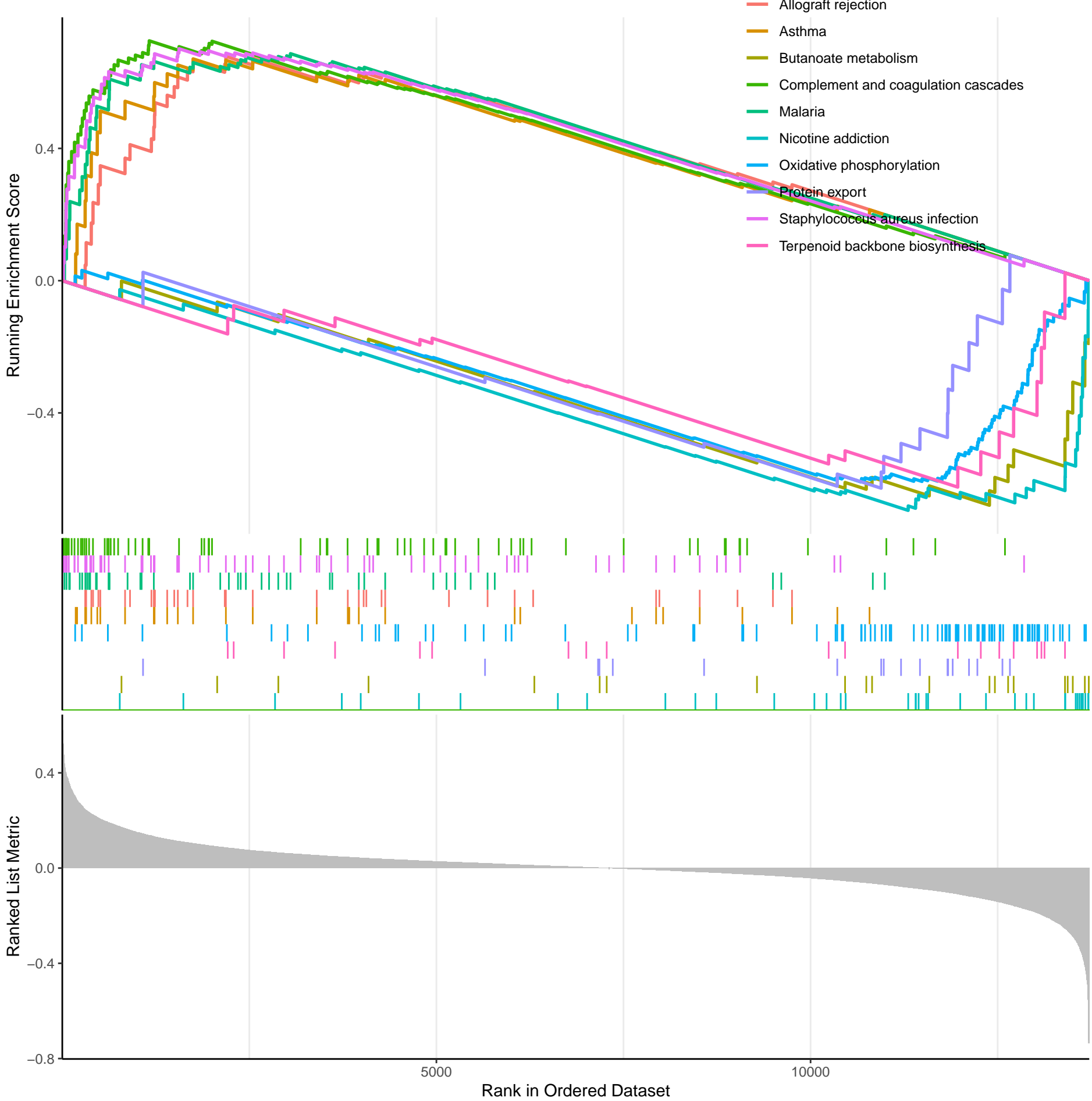

Supplement: Supplementary file 1 — Supplementary Information. [file 41598_2023_43595_MOESM1_ESM.zip › row data/Test group raw figure/ANTXR2_all_GSEA.pdf]

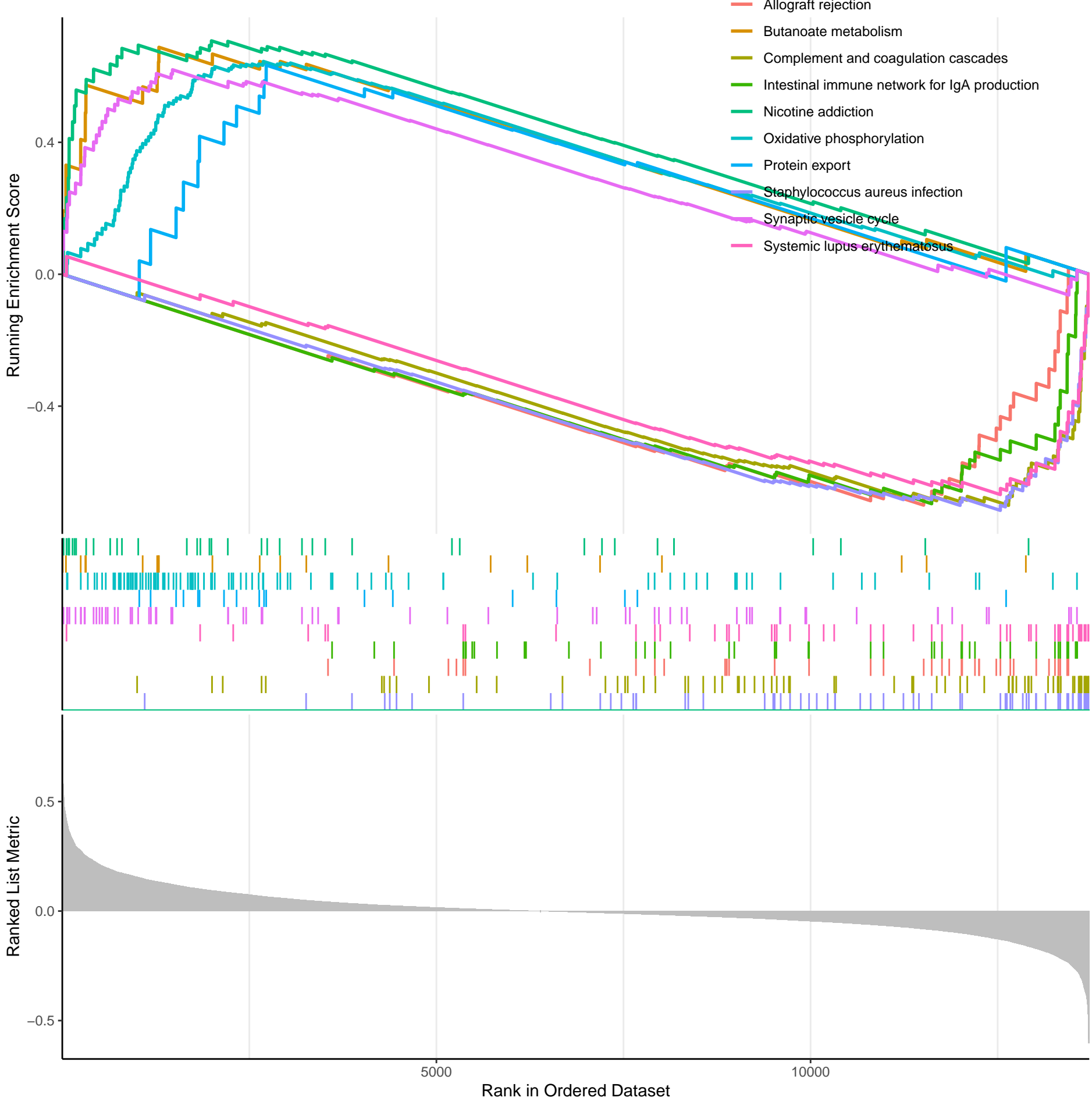

Supplement: Supplementary file 1 — Supplementary Information. [file 41598_2023_43595_MOESM1_ESM.zip › row data/Test group raw figure/BDNF_all_GSEA.pdf]

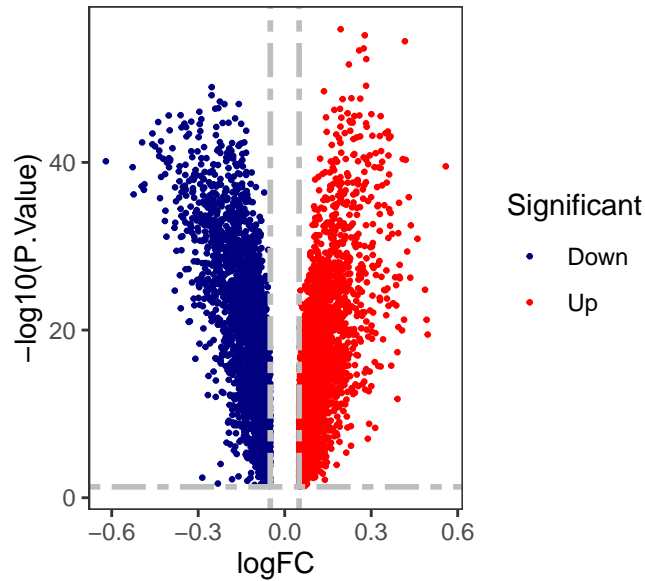

Supplement: Supplementary file 1 — Supplementary Information. [file 41598_2023_43595_MOESM1_ESM.zip › row data/Test group raw figure/consensus volcano.pdf]

# Gene dendrogram and module colors

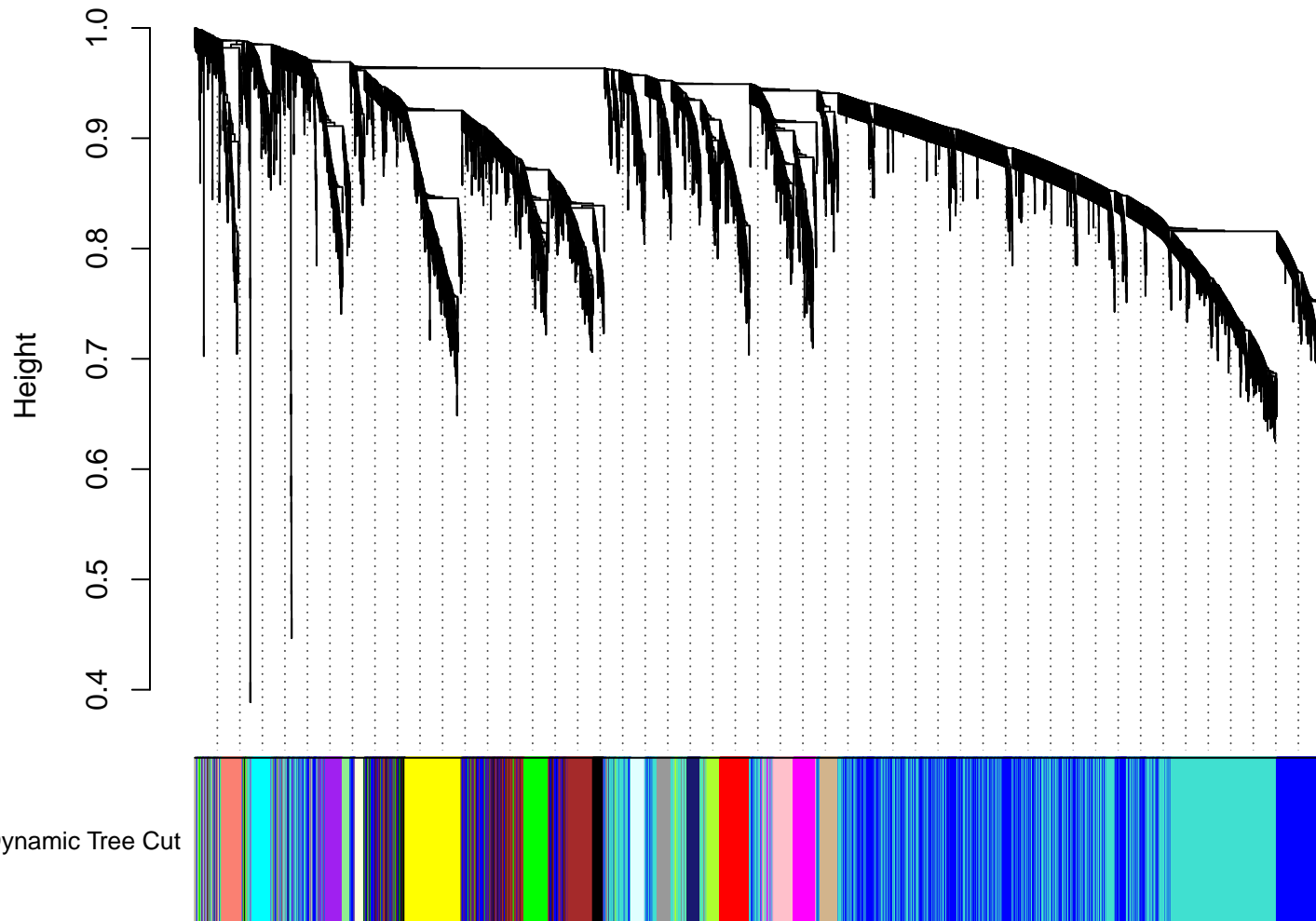

Supplement: Supplementary file 1 — Supplementary Information. [file 41598_2023_43595_MOESM1_ESM.zip › row data/Test group raw figure/Dynamic Tree Cut.pdf]

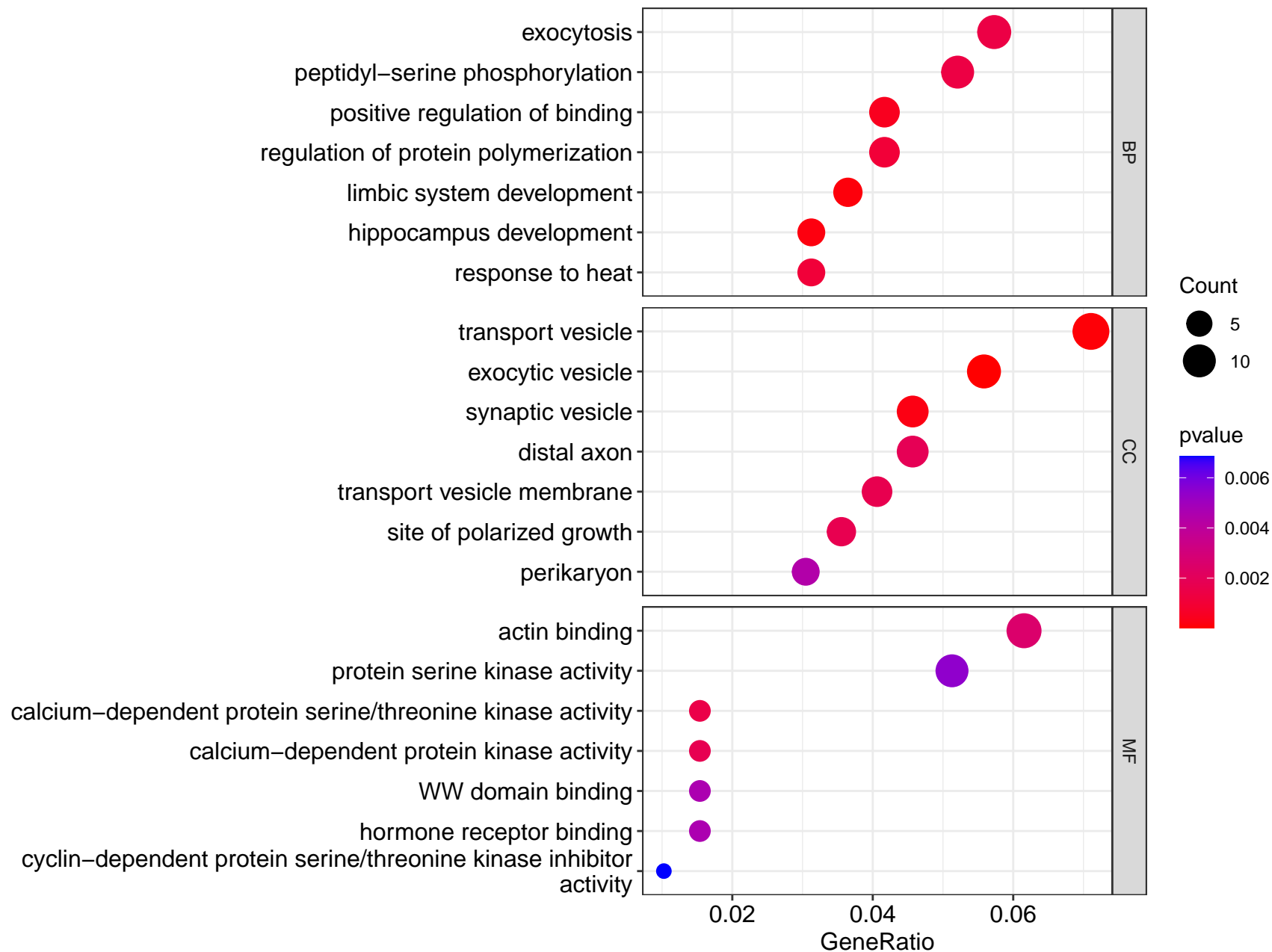

Supplement: Supplementary file 1 — Supplementary Information. [file 41598_2023_43595_MOESM1_ESM.zip › row data/Test group raw figure/GO_bubble.pdf]

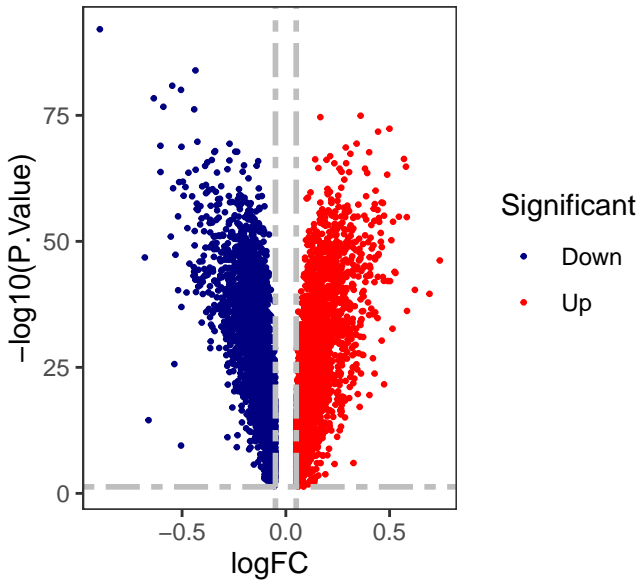

Supplement: Supplementary file 1 — Supplementary Information. [file 41598_2023_43595_MOESM1_ESM.zip › row data/Test group raw figure/GSE33000 volcano.pdf]

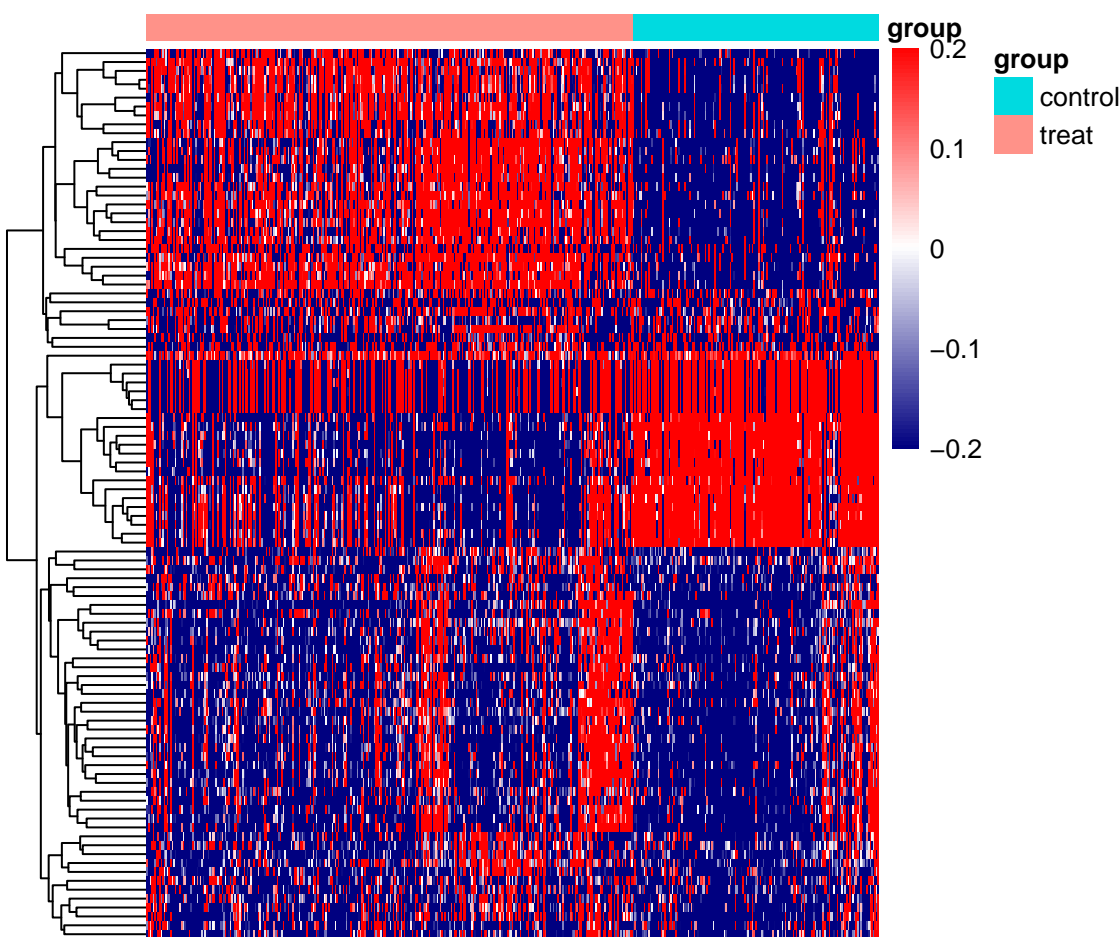

Supplement: Supplementary file 1 — Supplementary Information. [file 41598_2023_43595_MOESM1_ESM.zip › row data/Test group raw figure/heatmap.pdf]

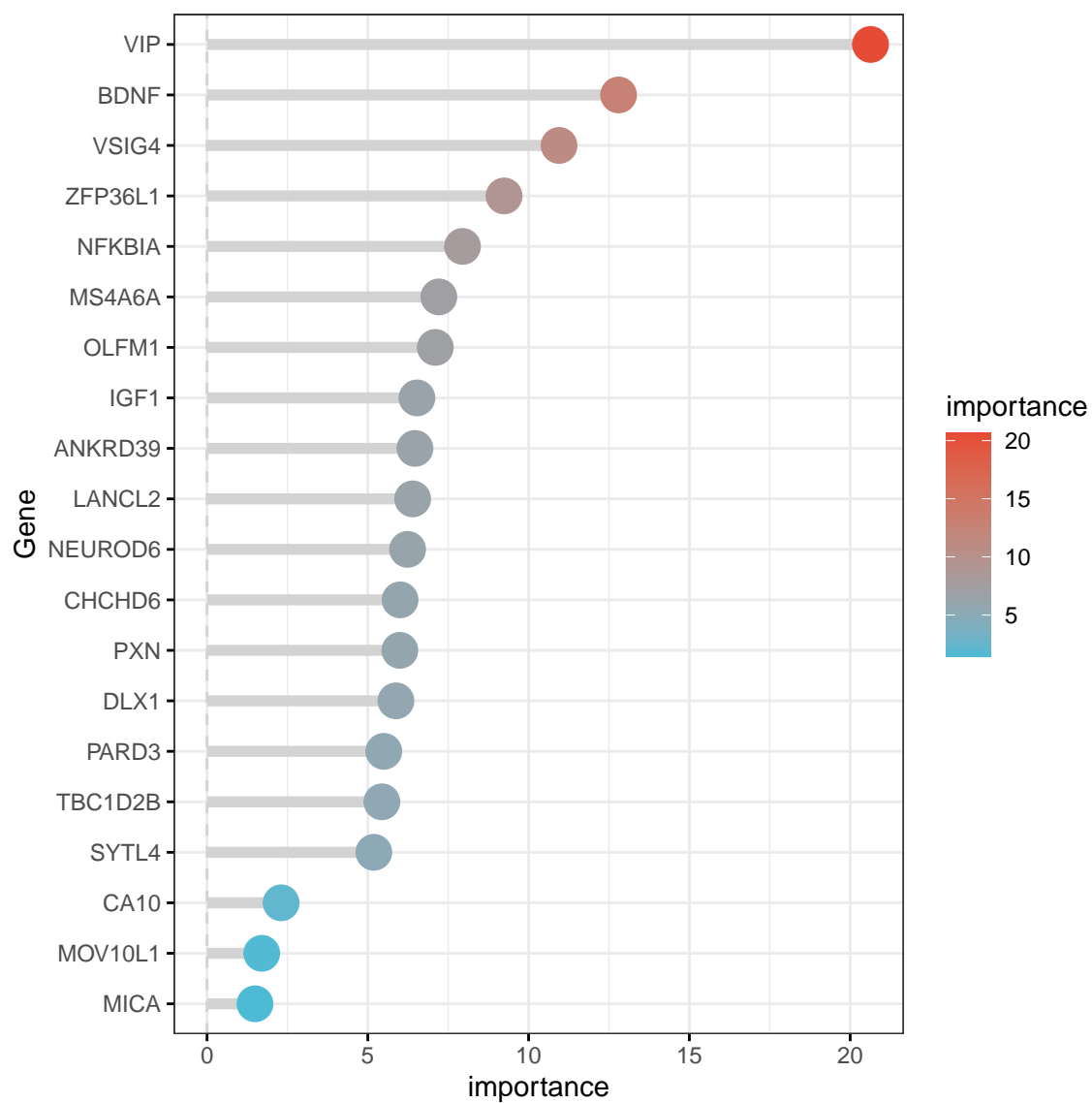

Supplement: Supplementary file 1 — Supplementary Information. [file 41598_2023_43595_MOESM1_ESM.zip › row data/Test group raw figure/importance.pdf]

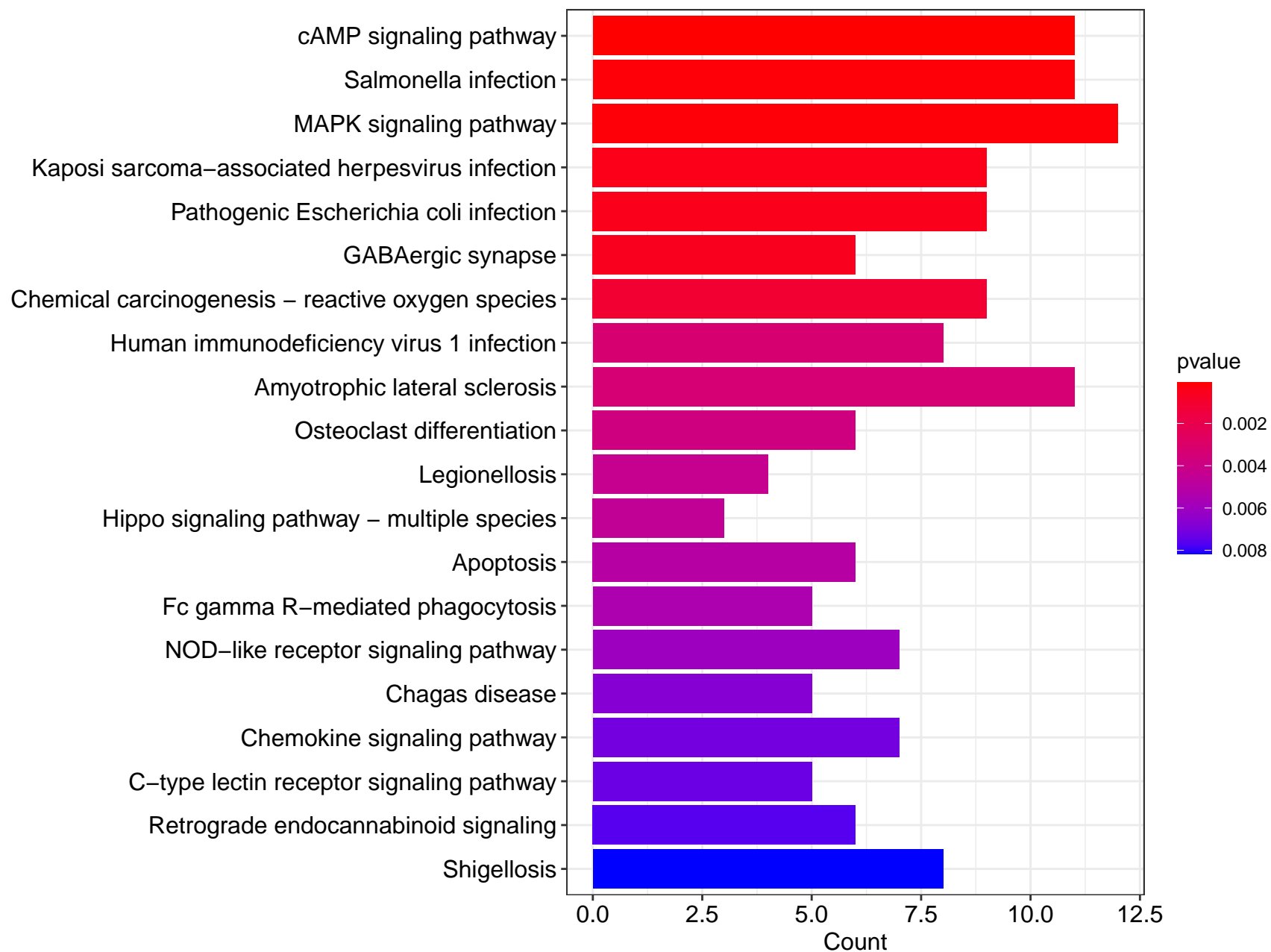

Supplement: Supplementary file 1 — Supplementary Information. [file 41598_2023_43595_MOESM1_ESM.zip › row data/Test group raw figure/KEGG_barplot.pdf]

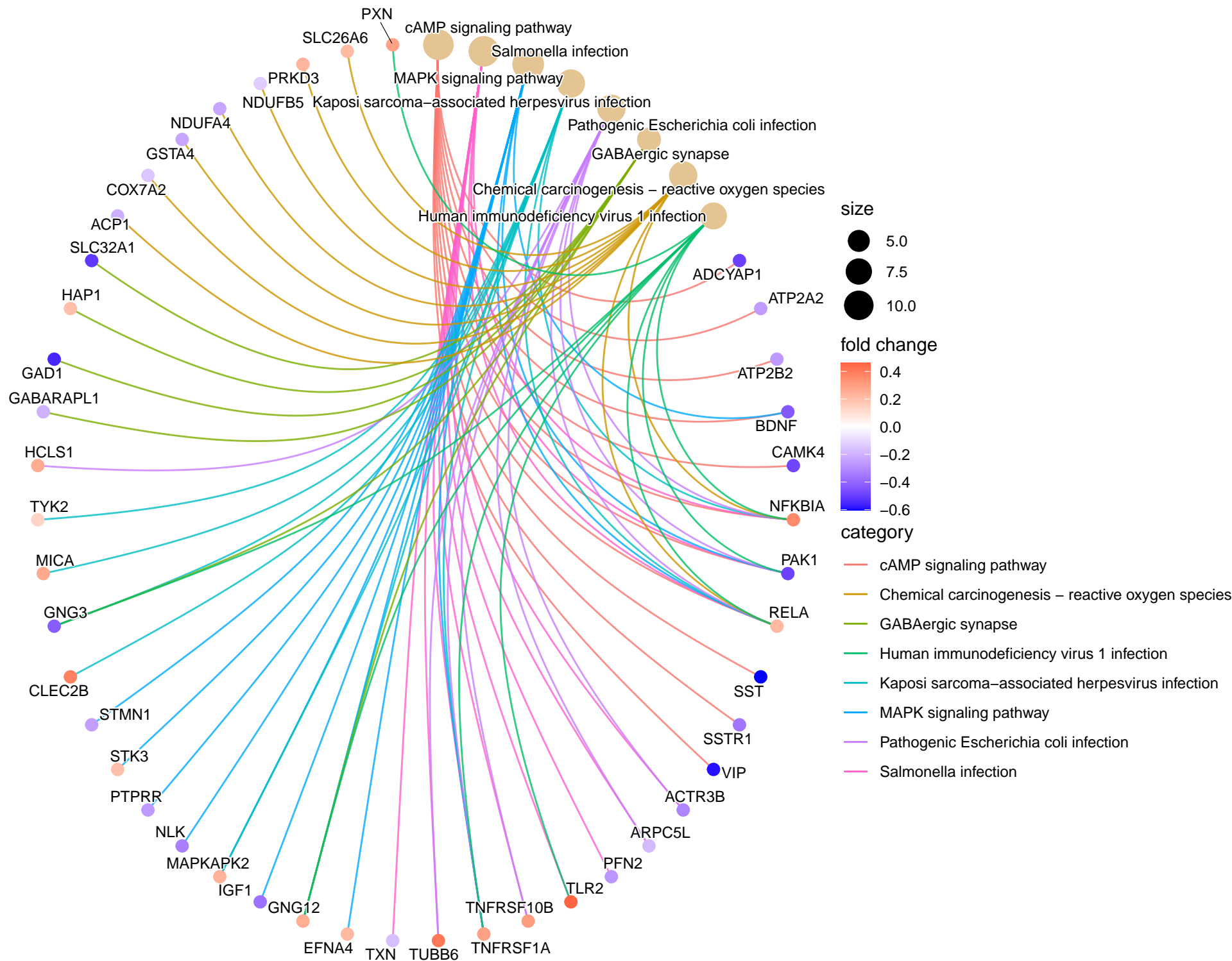

Supplement: Supplementary file 1 — Supplementary Information. [file 41598_2023_43595_MOESM1_ESM.zip › row data/Test group raw figure/KEGG_cnet.pdf]

Binomial Deviance

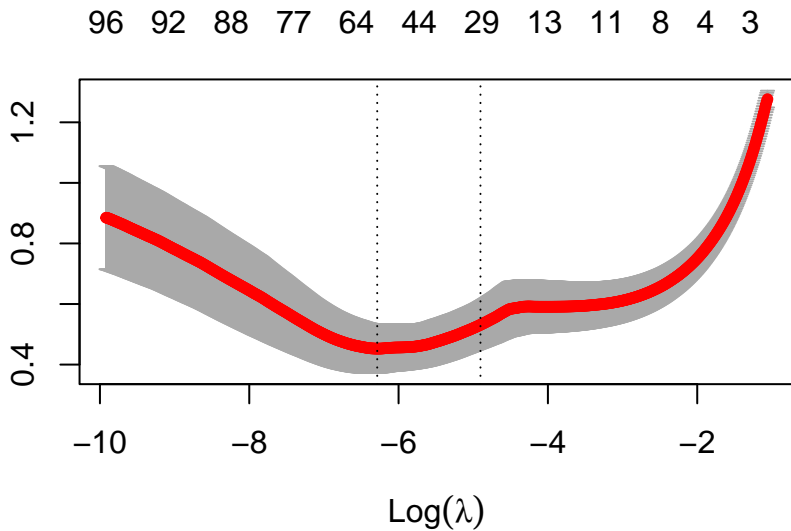

Supplement: Supplementary file 1 — Supplementary Information. [file 41598_2023_43595_MOESM1_ESM.zip › row data/Test group raw figure/lasso-cifit.pdf]

Coefficients

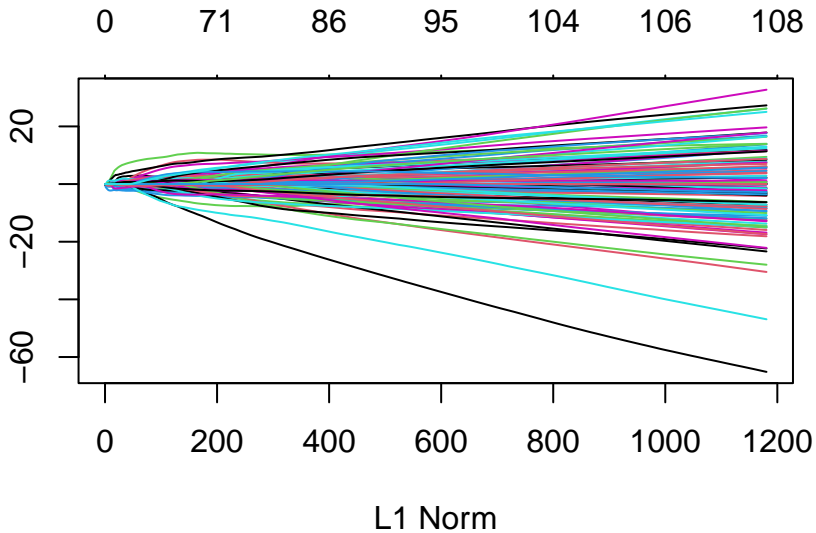

Supplement: Supplementary file 1 — Supplementary Information. [file 41598_2023_43595_MOESM1_ESM.zip › row data/Test group raw figure/model-lasso.pdf]

Module–trait relationships

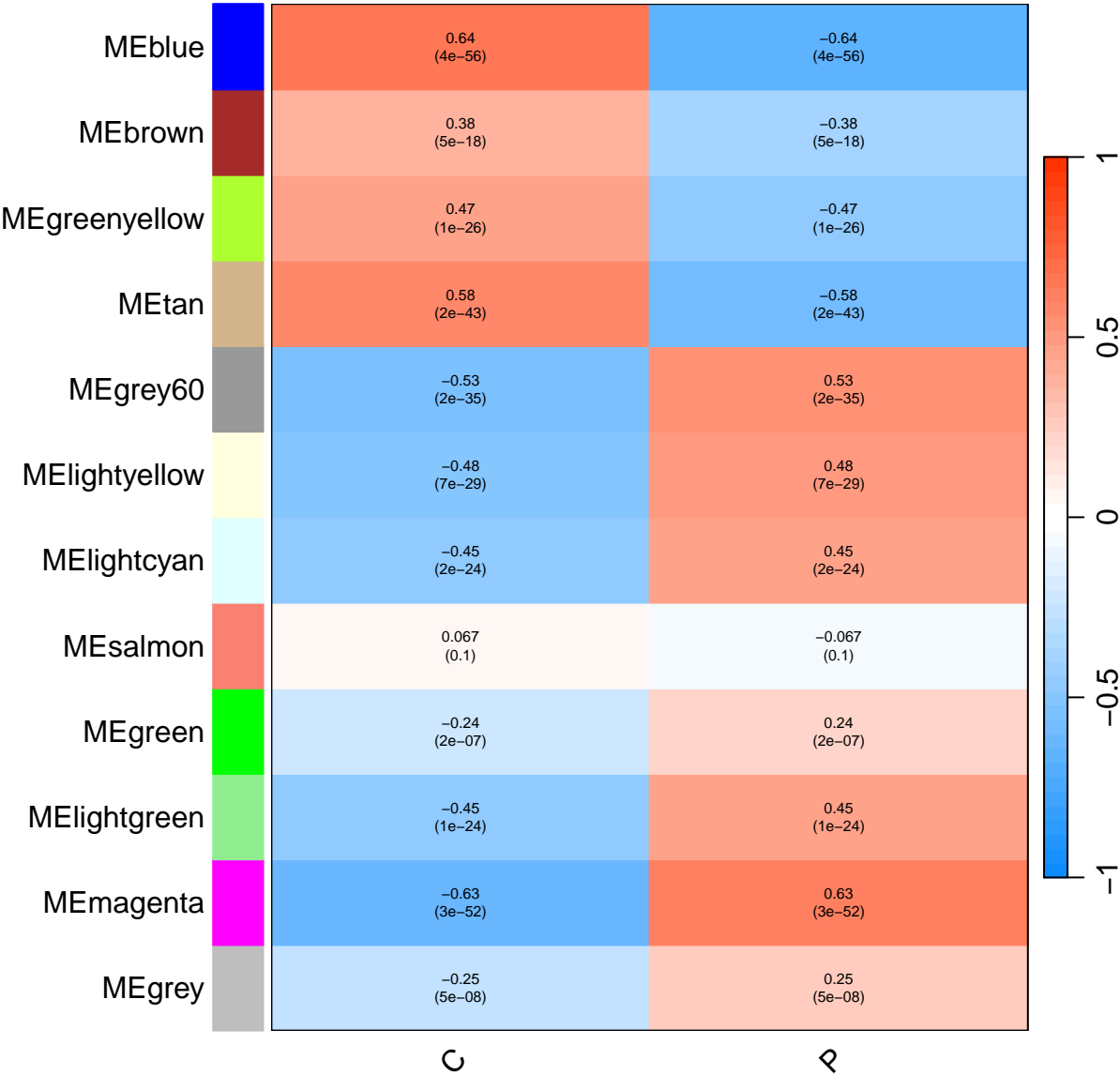

Supplement: Supplementary file 1 — Supplementary Information. [file 41598_2023_43595_MOESM1_ESM.zip › row data/Test group raw figure/Module-trait relationships.pdf]

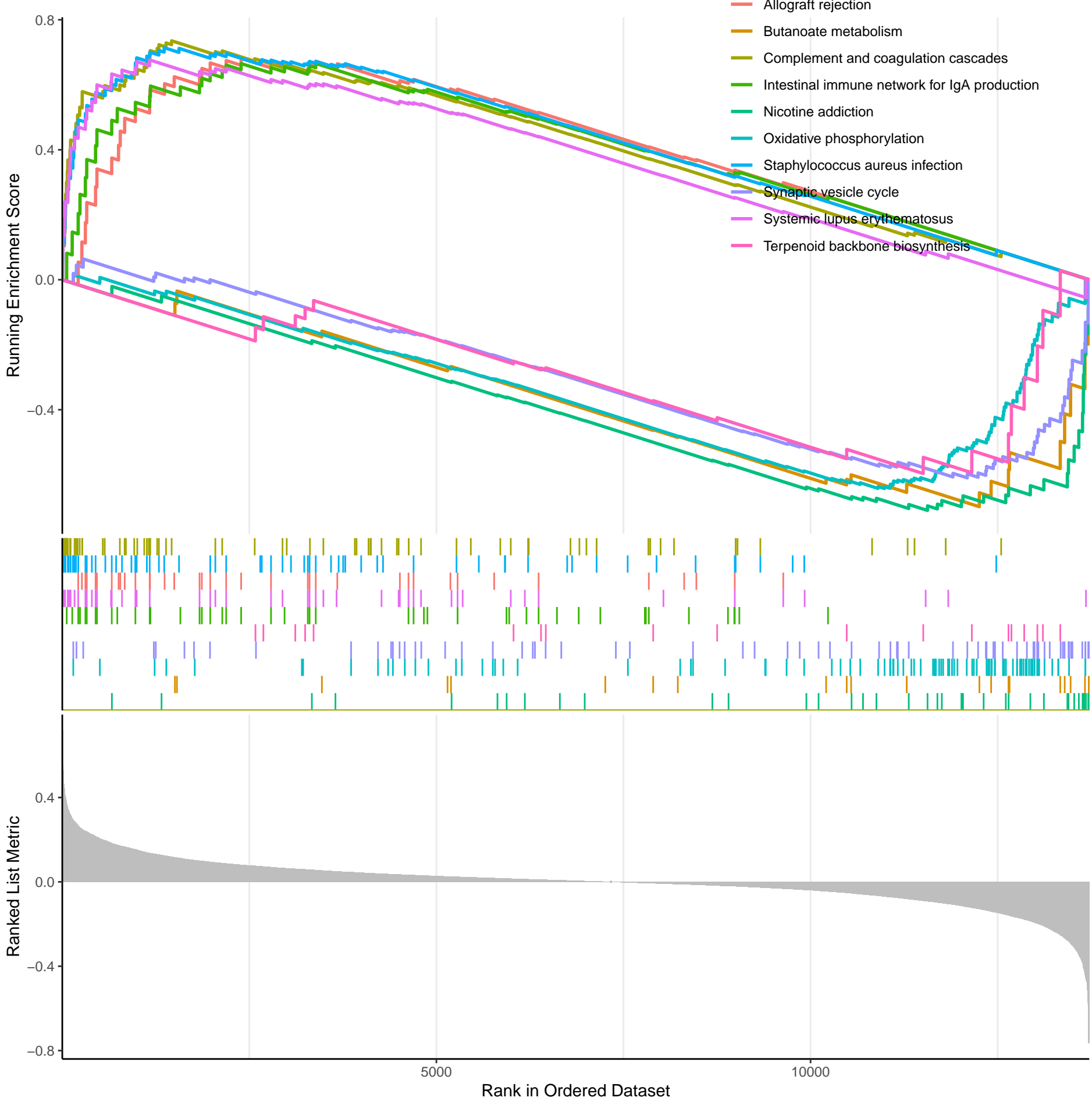

Supplement: Supplementary file 1 — Supplementary Information. [file 41598_2023_43595_MOESM1_ESM.zip › row data/Test group raw figure/MOV10L1_all_GSEA.pdf]

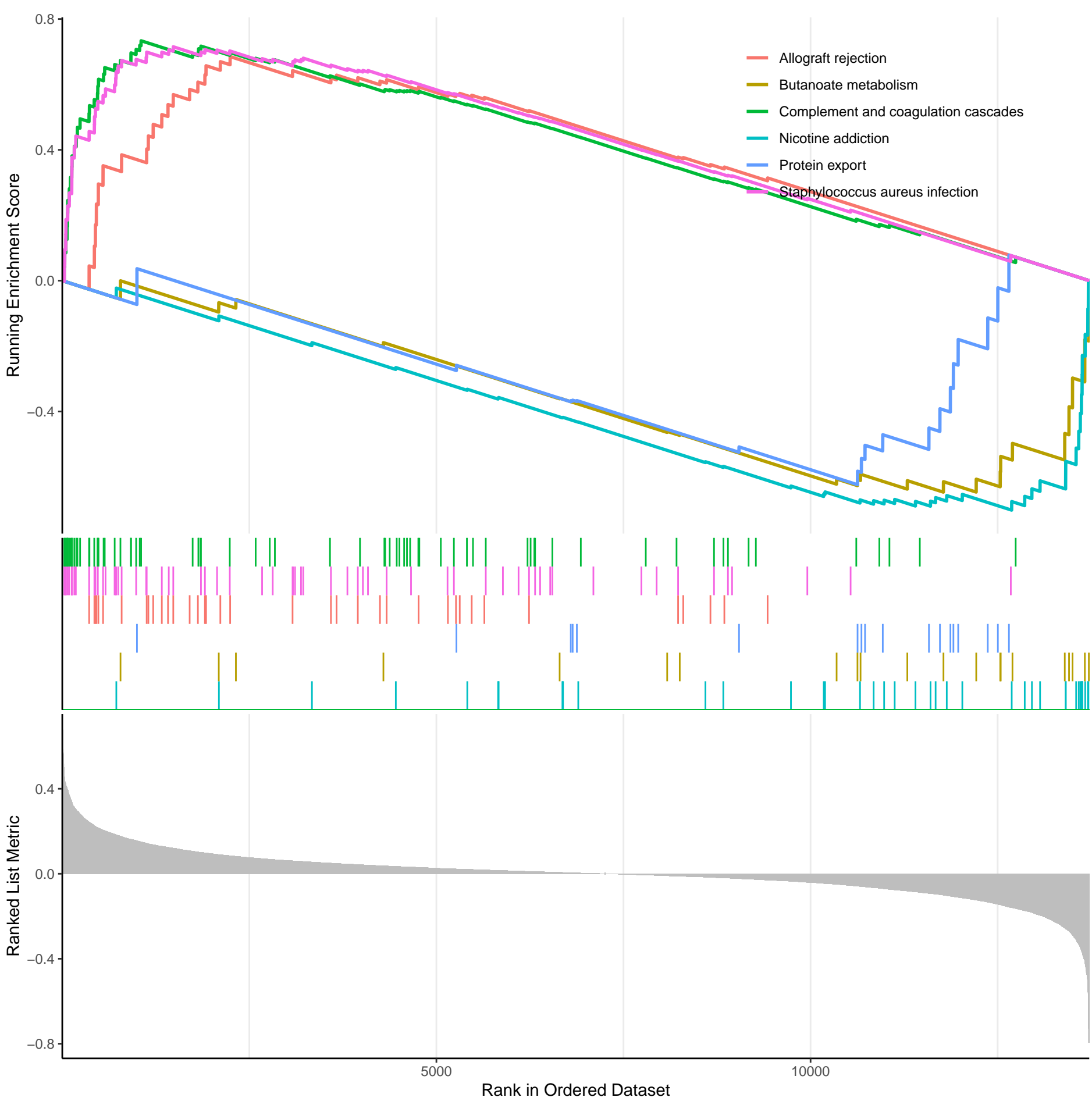

Supplement: Supplementary file 1 — Supplementary Information. [file 41598_2023_43595_MOESM1_ESM.zip › row data/Test group raw figure/NFKBIA_all_GSEA.pdf]

Points

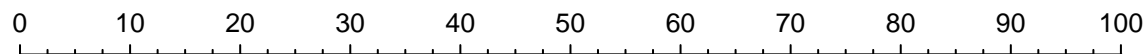

ANTXR2

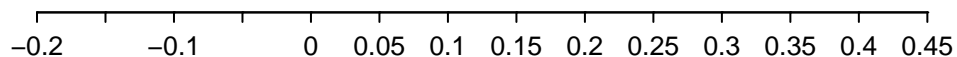

BDNF

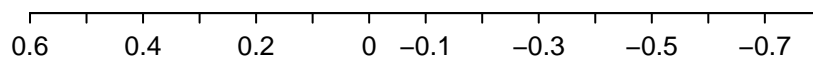

NFKBIA

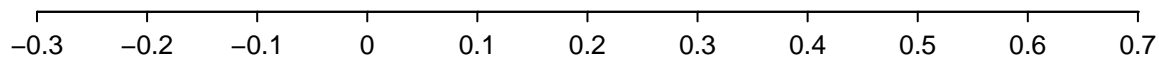

MOV10L1

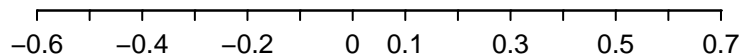

Total Points

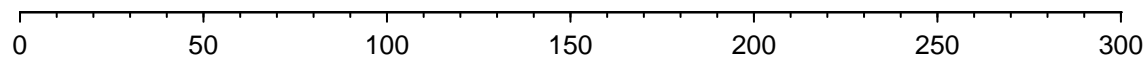

Linear Predictor

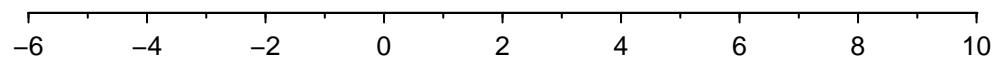

Supplement: Supplementary file 1 — Supplementary Information. [file 41598_2023_43595_MOESM1_ESM.zip › row data/Test group raw figure/nomogram.pdf]

**nomoscore**

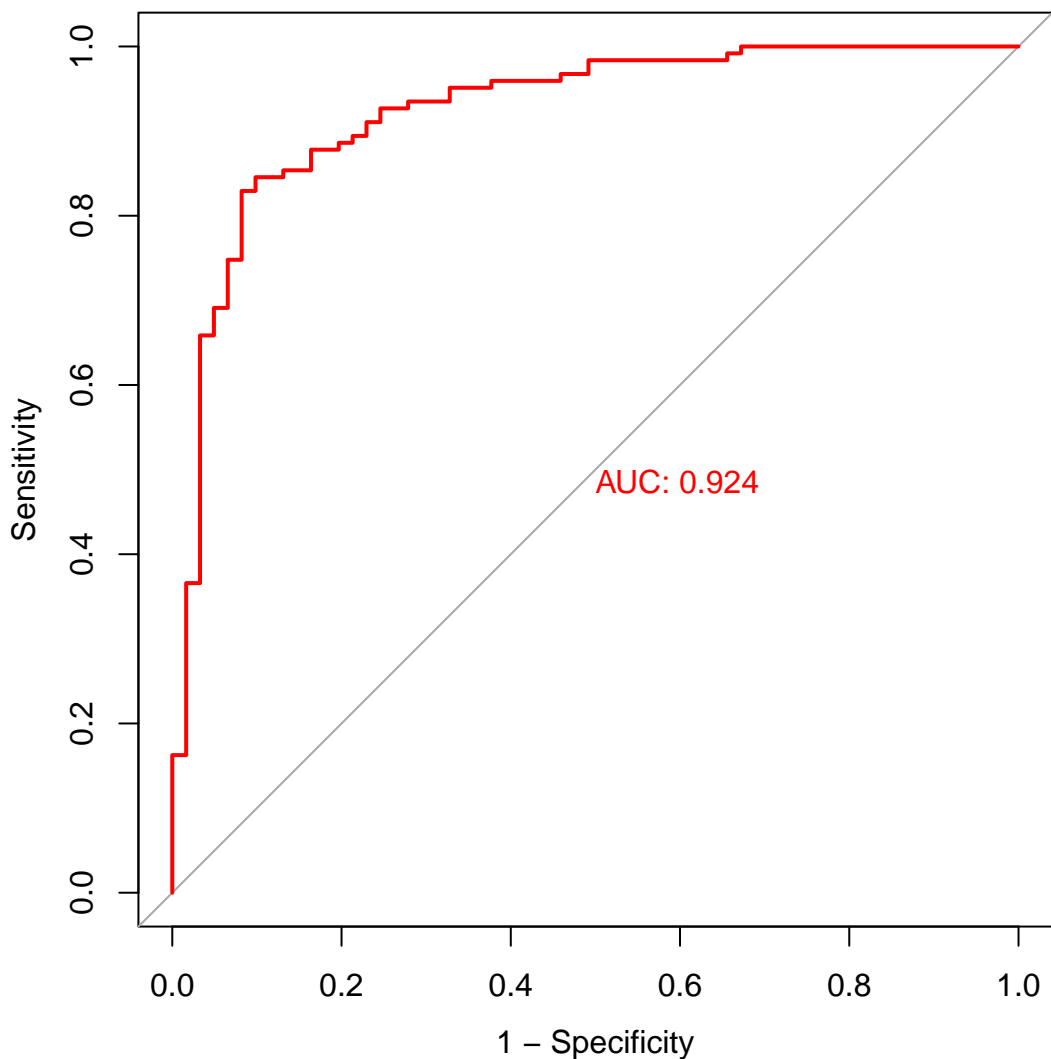

Supplement: Supplementary file 1 — Supplementary Information. [file 41598_2023_43595_MOESM1_ESM.zip › row data/Test group raw figure/nomoscore_Ptest.pdf]

nomoscore

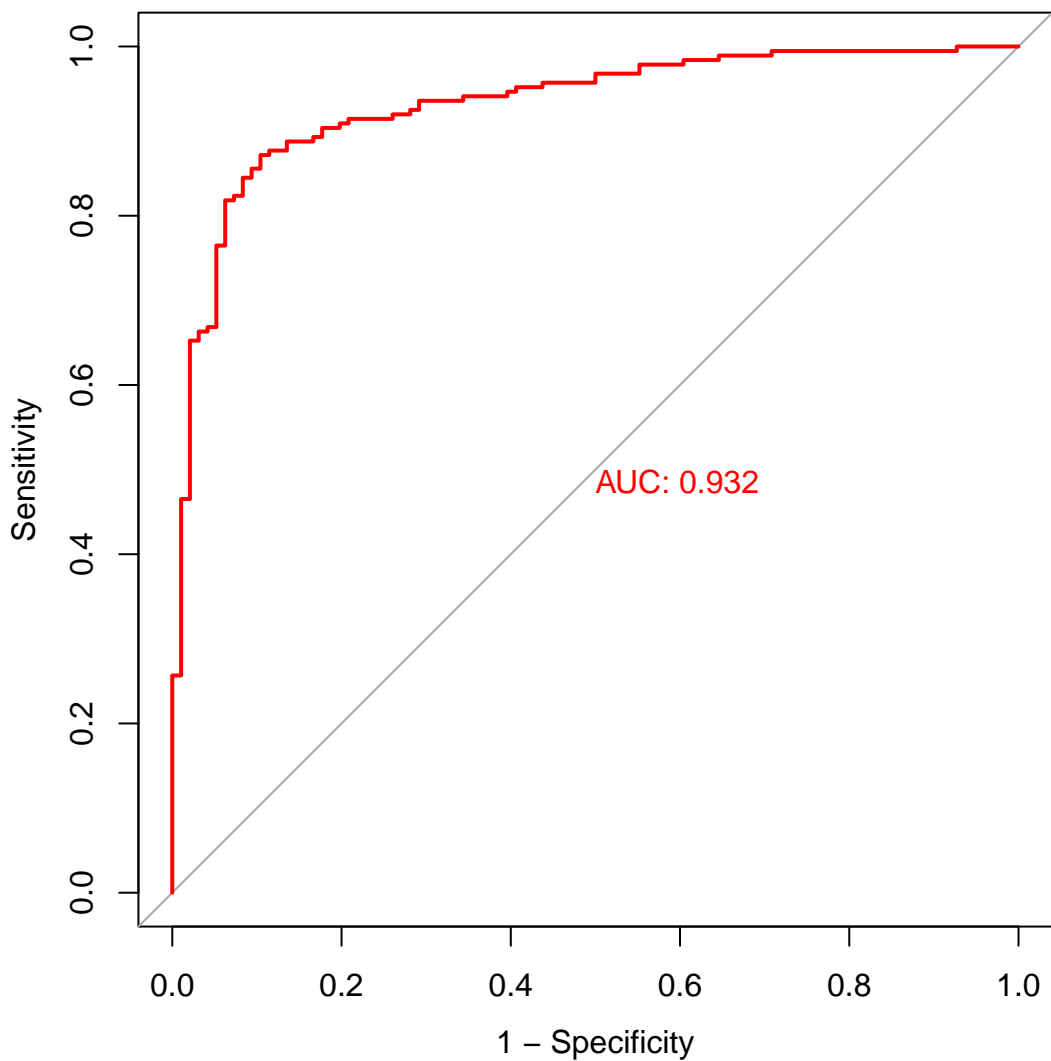

Supplement: Supplementary file 1 — Supplementary Information. [file 41598_2023_43595_MOESM1_ESM.zip › row data/Test group raw figure/nomoscore_Ptrain.pdf]

**Module membership vs. gene significance**  
**cor=0.77,  $p < 1e-200$**

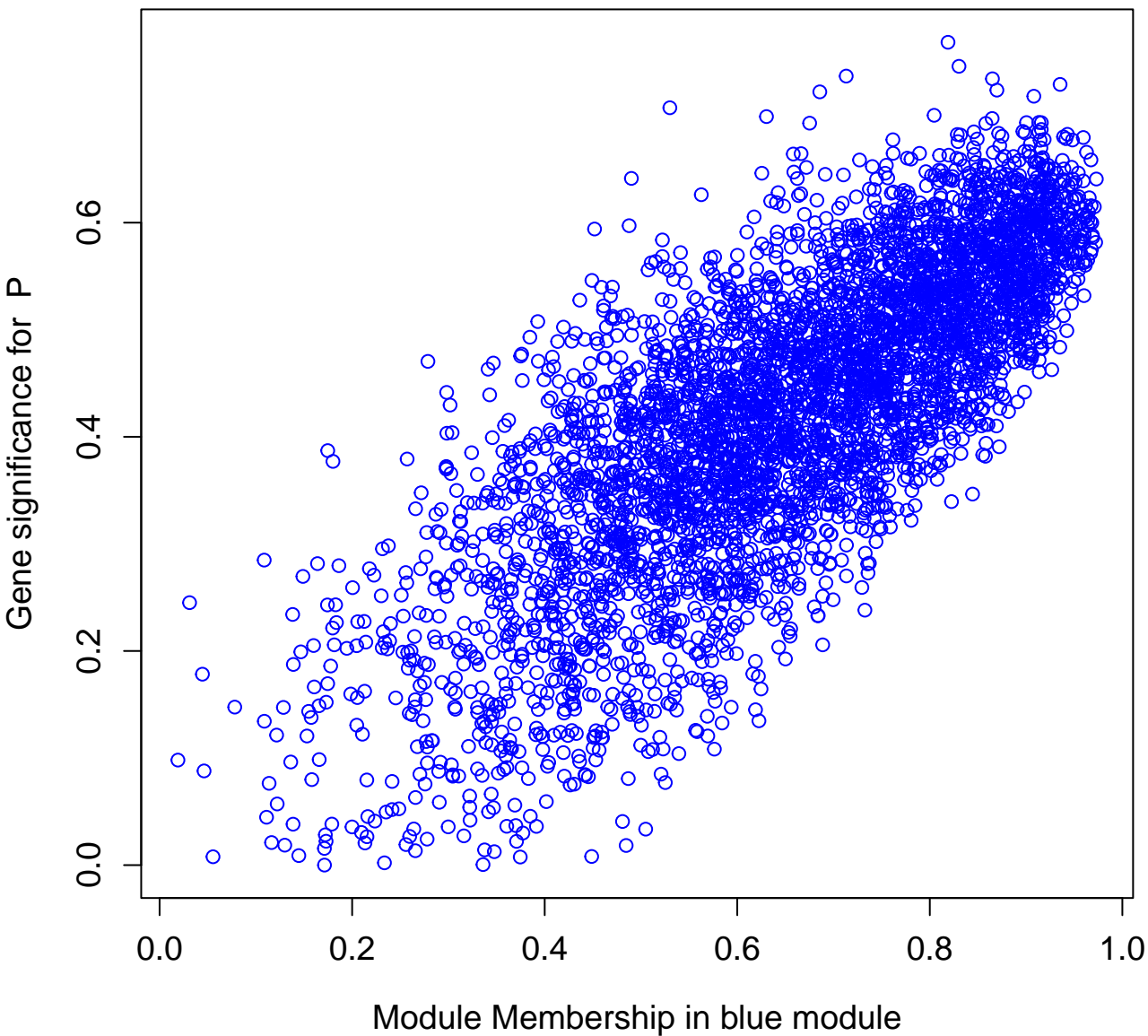

Supplement: Supplementary file 1 — Supplementary Information. [file 41598_2023_43595_MOESM1_ESM.zip › row data/Test group raw figure/P_blue_Module membership vs gene significance.pdf]

**Module membership vs. gene significance**  
**cor=0.84, p=3.3e-103**

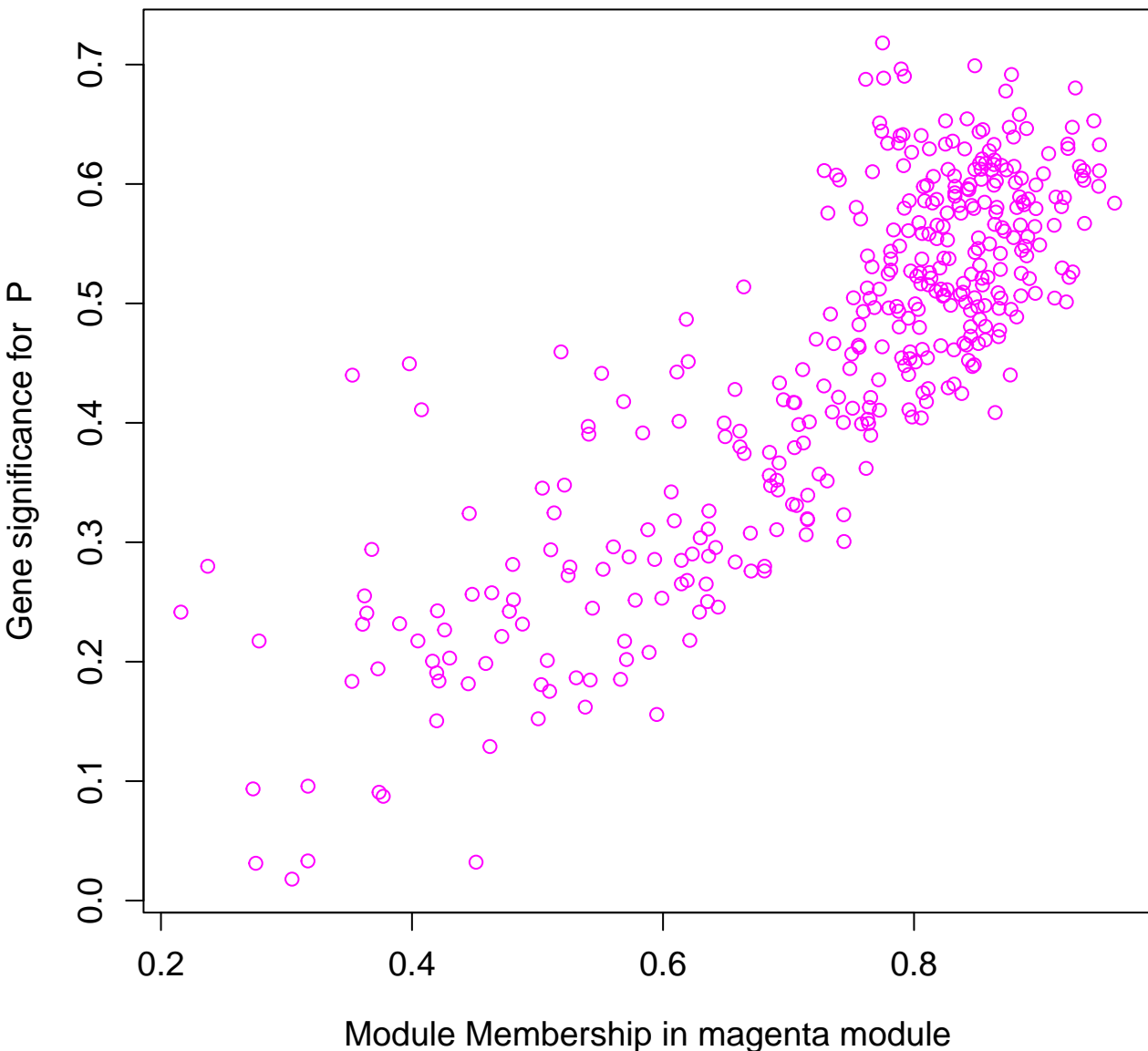

Supplement: Supplementary file 1 — Supplementary Information. [file 41598_2023_43595_MOESM1_ESM.zip › row data/Test group raw figure/P_magenta_Module membership vs gene significance.pdf]

### Scale independence

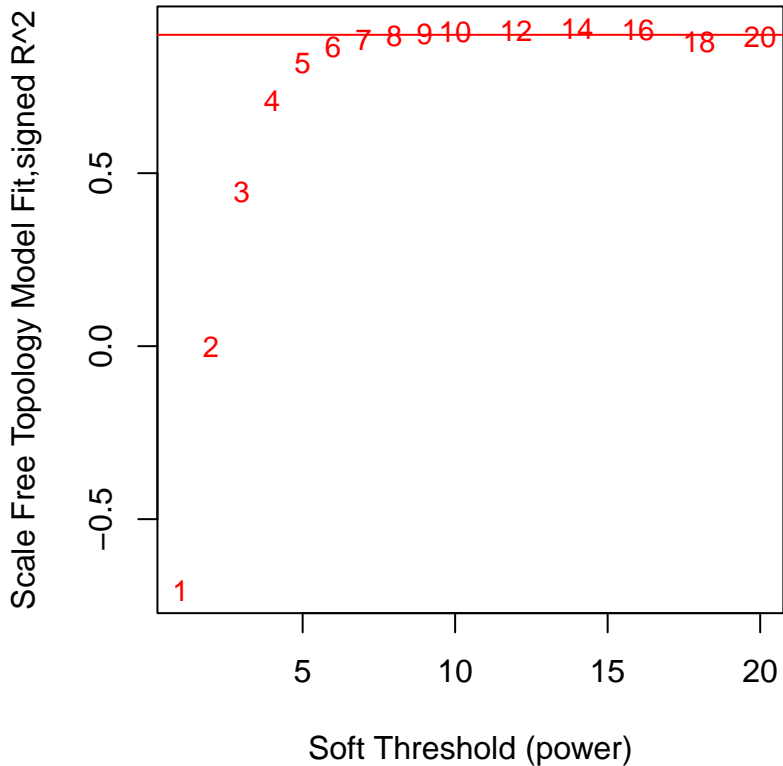

### Mean connectivity

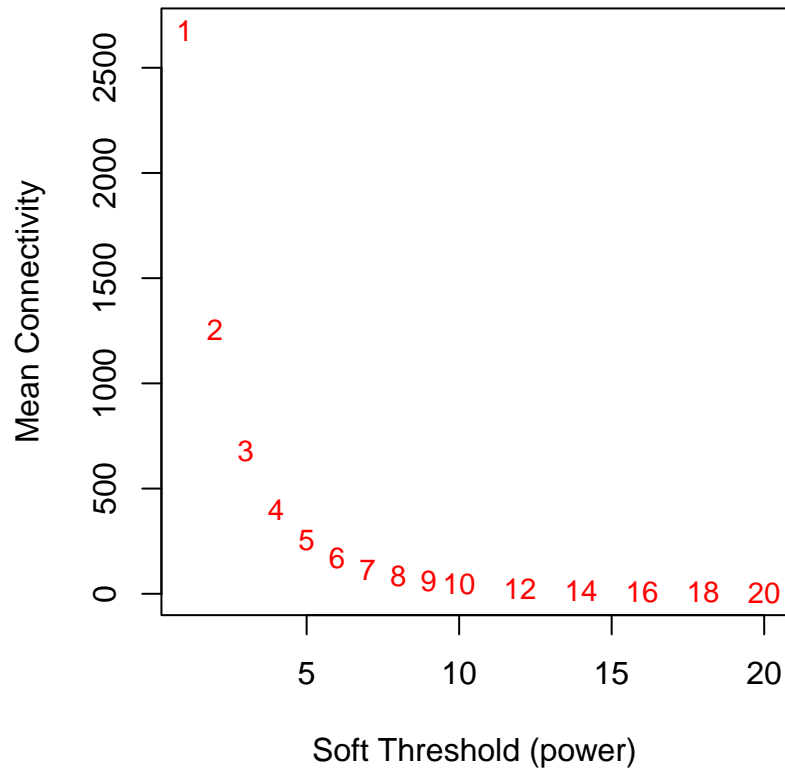

Supplement: Supplementary file 1 — Supplementary Information. [file 41598_2023_43595_MOESM1_ESM.zip › row data/Test group raw figure/Scale independence.pdf]

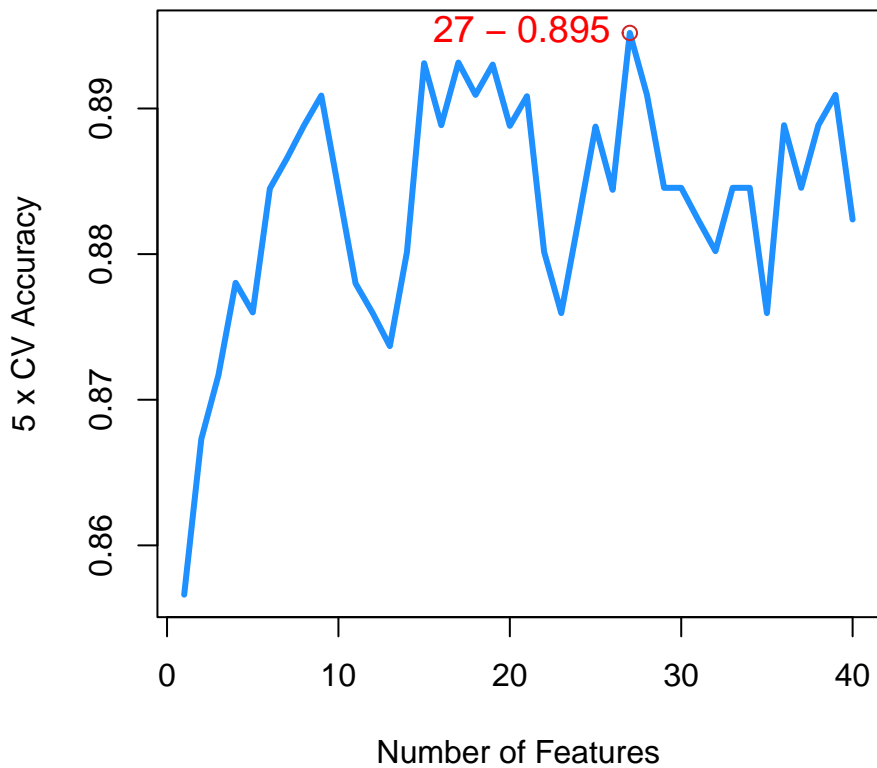

Supplement: Supplementary file 1 — Supplementary Information. [file 41598_2023_43595_MOESM1_ESM.zip › row data/Test group raw figure/svm-accuracy.pdf]

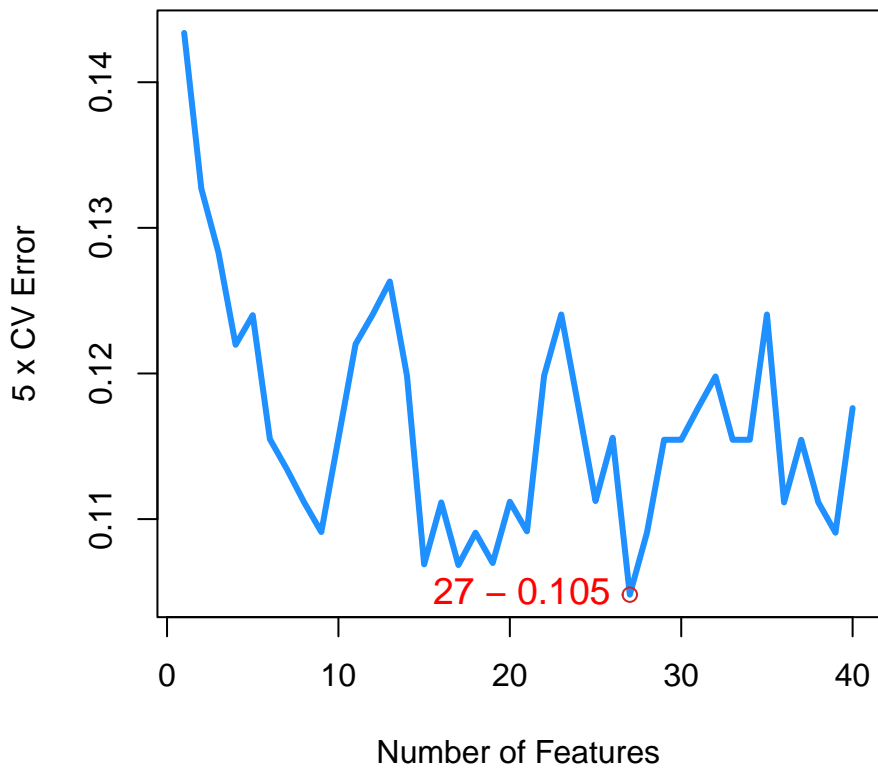

Supplement: Supplementary file 1 — Supplementary Information. [file 41598_2023_43595_MOESM1_ESM.zip › row data/Test group raw figure/svm-error.pdf]

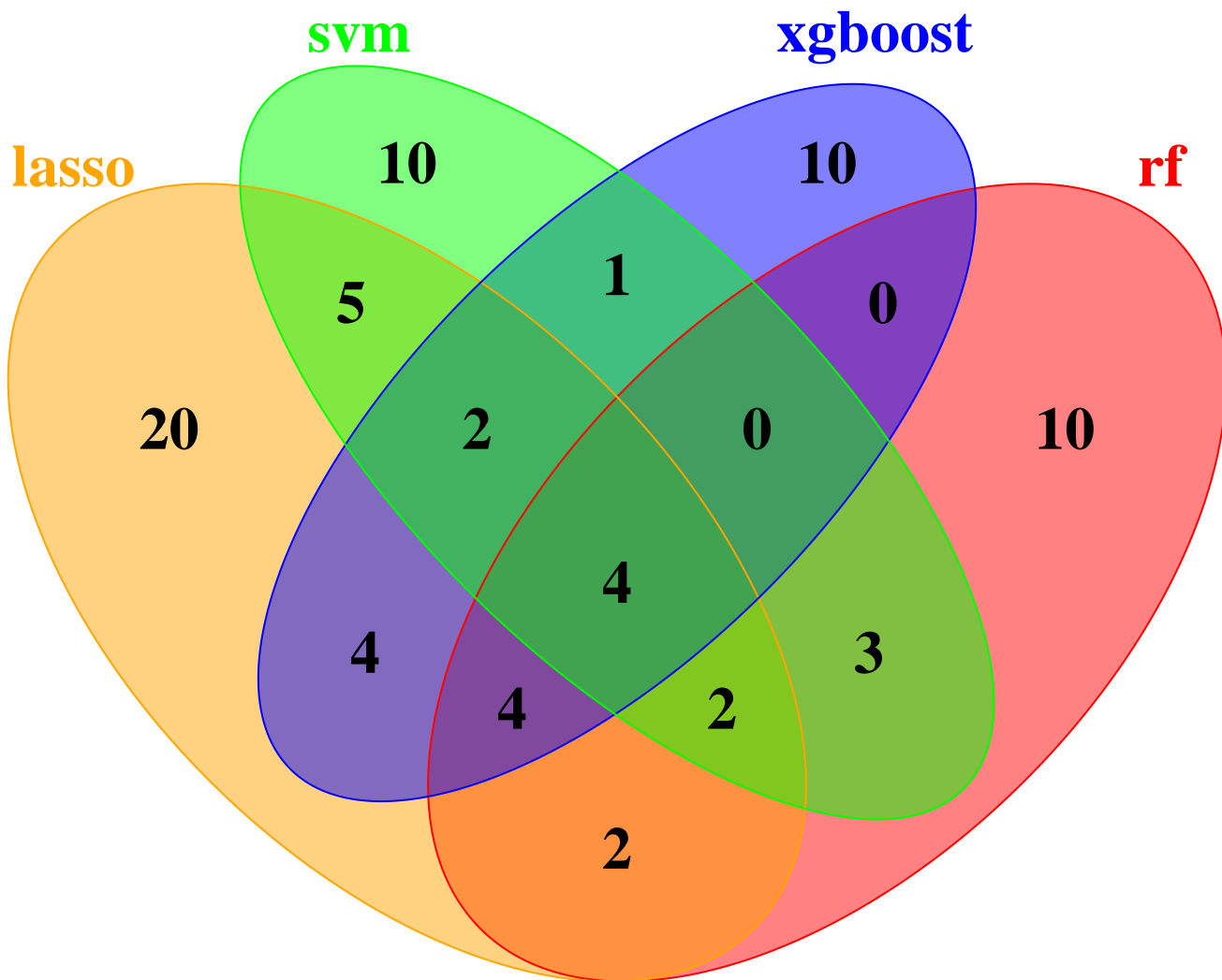

Supplement: Supplementary file 1 — Supplementary Information. [file 41598_2023_43595_MOESM1_ESM.zip › row data/Test group raw figure/venn.pdf]

# Feature importance

Features

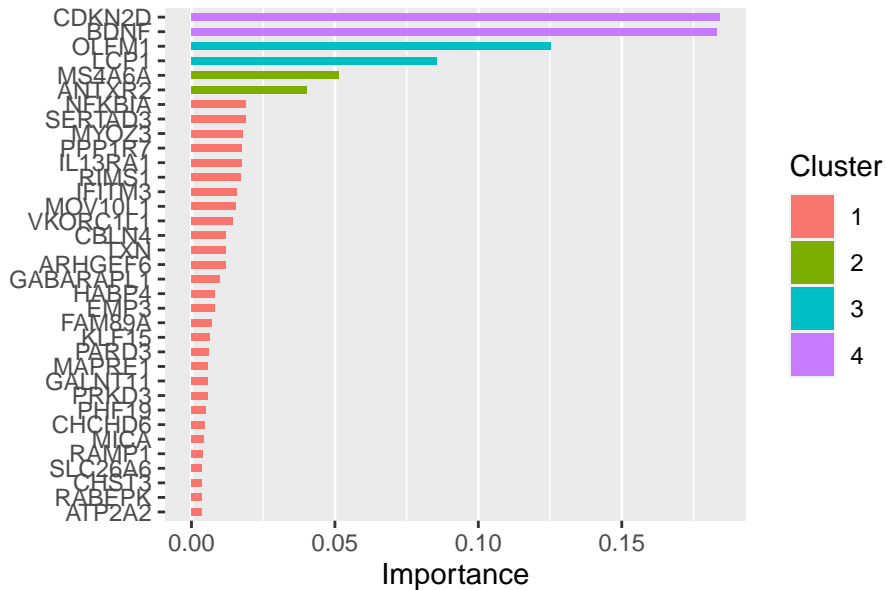

Supplement: Supplementary file 1 — Supplementary Information. [file 41598_2023_43595_MOESM1_ESM.zip › row data/Test group raw figure/xgboost.pdf]

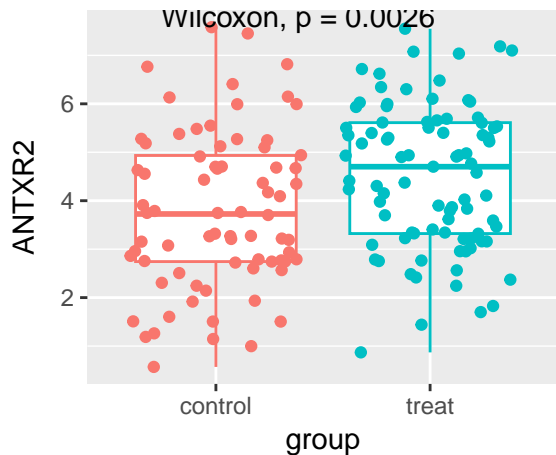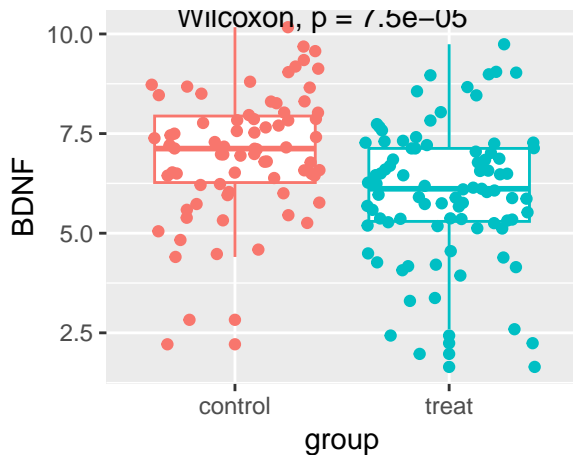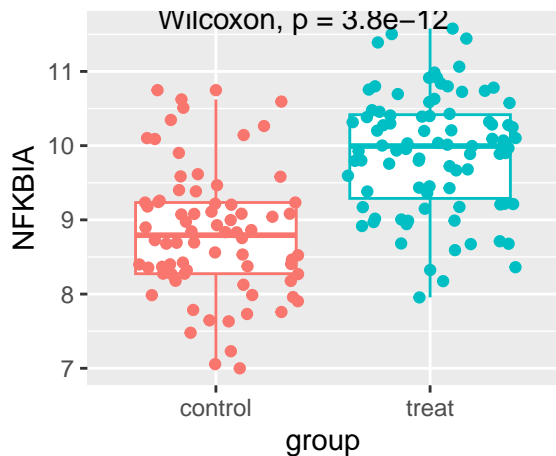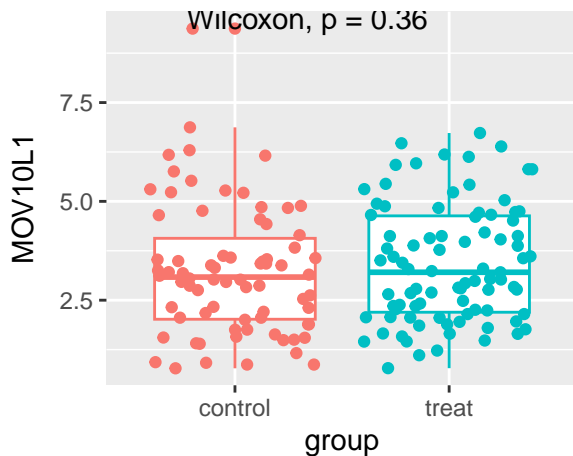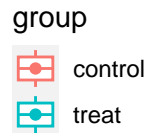

Supplement: Supplementary file 1 — Supplementary Information. [file 41598_2023_43595_MOESM1_ESM.zip › row data/Validation group raw figure/four genes.pdf]

nomoscore

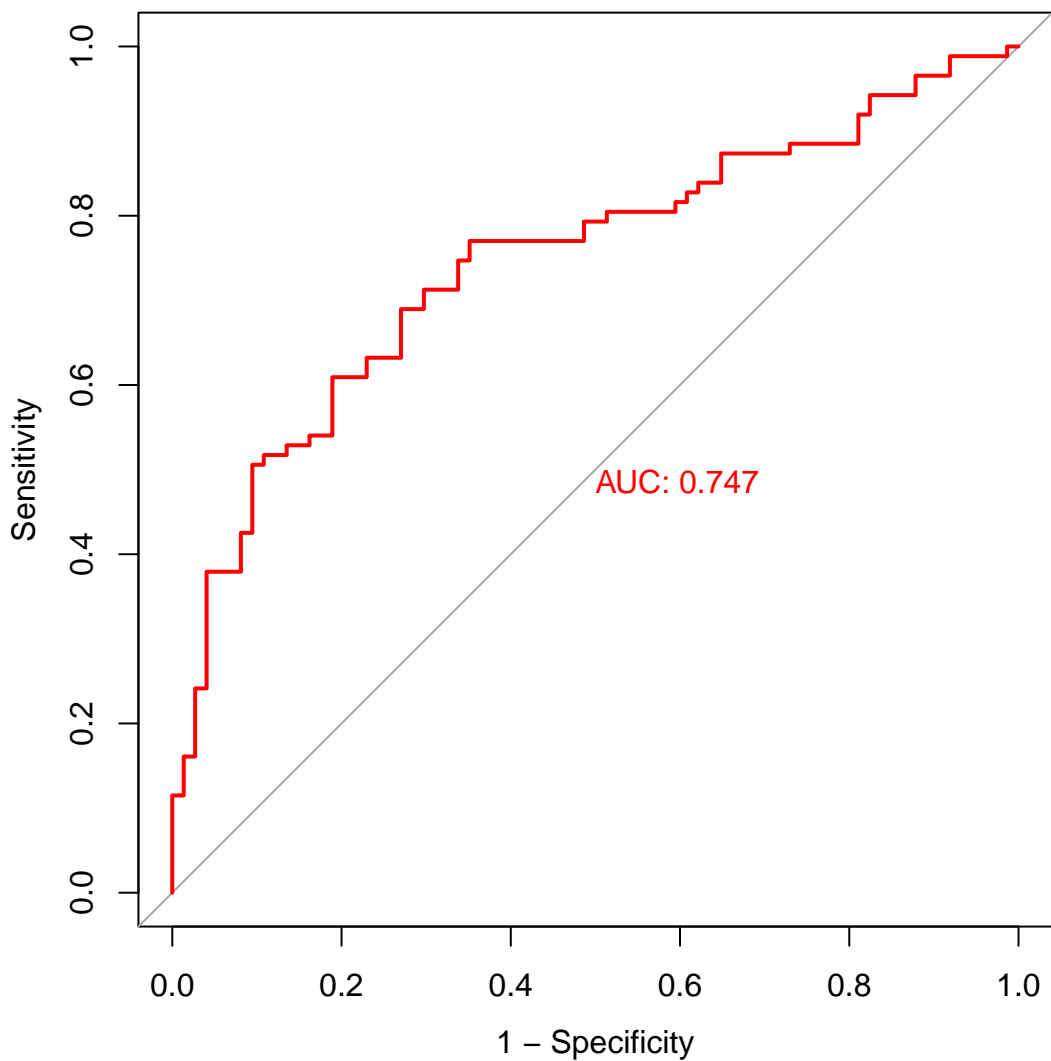

Supplement: Supplementary file 1 — Supplementary Information. [file 41598_2023_43595_MOESM1_ESM.zip › row data/Validation group raw figure/validation roc.pdf]

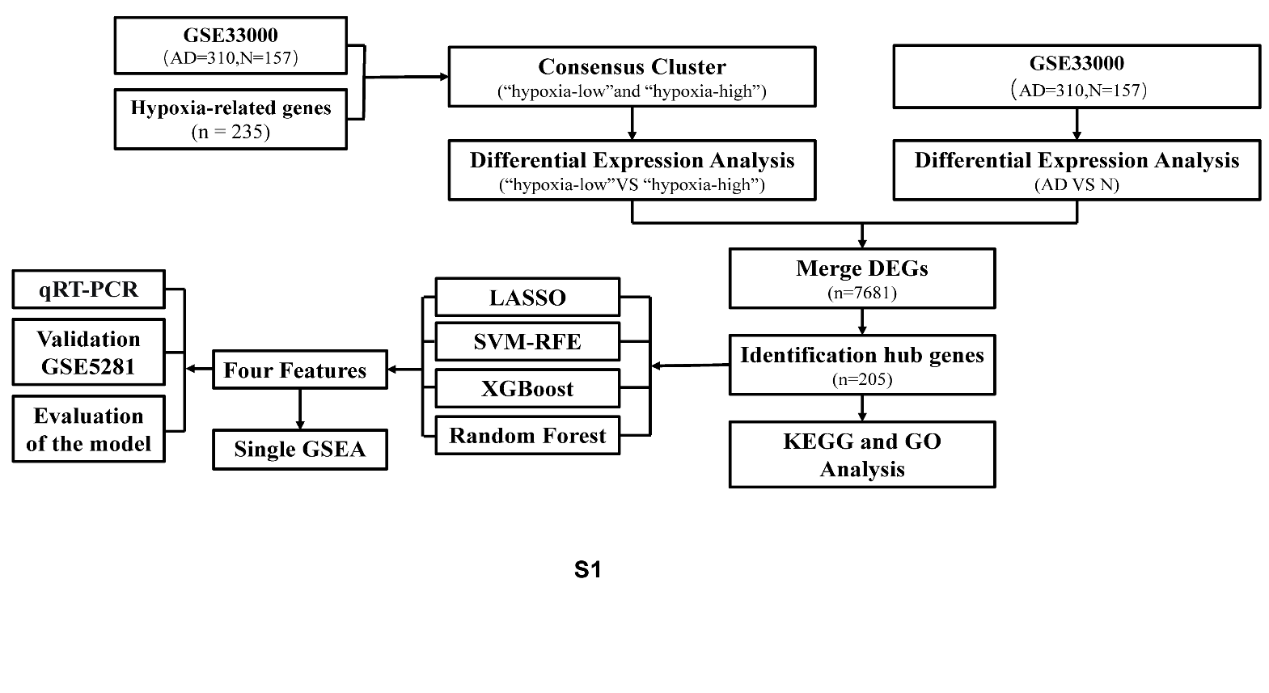


S1 **The overview of the whole study.**

Supplement: Supplementary file 2 — Supplementary Figure S1. [file 41598_2023_43595_MOESM2_ESM.docx]
